# Farey Trees Explain Sequential Effects in Choice Response Time

**DOI:** 10.3389/fphys.2021.611145

**Published:** 2021-03-17

**Authors:** Colin T. Annand, Sheila M. Fleming, John G. Holden

**Affiliations:** ^1^The Complexity Group, Department of Psychology, University of Cincinnati, Cincinnati, OH, United States; ^2^Department of Pharmaceutical Sciences, Northeast Ohio Medical University, Rootstown Township, OH, United States

**Keywords:** sequential effects, oscillatory entrainment, choice response time modeling, bimanual coordination, cognitive dynamics, nonlinear dynamics

## Abstract

The latencies of successive two-alternative, forced-choice response times display intricately patterned sequential effects, or dependencies. They vary as a function of particular trial-histories, and in terms of the order and identity of previously presented stimuli and registered responses. This article tests a novel hypothesis that sequential effects are governed by dynamic principles, such as those entailed by a discrete sine-circle map adaptation of the Haken Kelso Bunz (HKB) bimanual coordination model. The model explained the sequential effects expressed in two classic sequential dependency data sets. It explained the rise of a repetition advantage, the acceleration of repeated affirmative responses, in tasks with faster paces. Likewise, the model successfully predicted an alternation advantage, the acceleration of interleaved affirmative and negative responses, when a task’s pace slows and becomes more variable. Detailed analyses of five studies established oscillatory influences on sequential effects in the context of balanced and biased trial presentation rates, variable pacing, progressive and differential cognitive loads, and dyadic performance. Overall, the empirical patterns revealed lawful oscillatory constraints governing sequential effects in the time-course and accuracy of performance across a broad continuum of recognition and decision activities.

## Introduction and Overview

Successively measured two-alternative forced-choice response time latencies often display sequential dependencies in their durations. This propensity is a long-recognized empirical puzzle in the decision-making literature (e.g., see Hyman, 1953; Laming, 1969; Schvaneveldt and Chase, 1969; Kornblum, 1973; Kirby, 1976; Treisman and Williams, 1984; Luce, 1986; Cho et al., 2002; Jentzsch and Sommer, 2002; Doshi et al., 2012; Jones et al., 2013; Zhang et al., 2014). Other things equal, successive repeats of targeted stimulus-response relations yield a progressive speeding in response times across successive trials. This tendency is called the repetition effect. On the other hand, sequences of un-targeted or negative responses tend to yield progressive response slowing. Response times for trial-histories that are composed of various permutations of affirmative and negative responses typically fall between these extremes. These core tendencies are generally most acute in simpler tasks with relatively short inter-trial-intervals (ITIs – also called response-stimulus intervals or RSIs), the blank-screen downtime between trials. However, if the task imposes relatively long ITIs (>500ms), then *alternating* strings of affirmative and negative trials often yield more potent progressive speeding, referred to as the alternation effect (Kirby, 1980).

Historically, the cognitive literature modeled sequential effects with Markov chains. Modern models emphasize Bayesian and connectionist approaches. They all successfully reproduce various aspects of the empirical trial-history patterns. The earliest models posited *post hoc* faciliatory cognitive mechanisms, primarily as an explanation for repetition effects (e.g., Laming, 1969). Higher-level strategic expectancy mechanisms were also posited as explanations for either repetition or alternation effects. By the mid-1970’s hybrid models combined dedicated expectancy and facilitation mechanisms (Kirby, 1980). Similarly, Luce (1986) reviewed Markov models that combined fast-guessing with short-term memory functions. By this time, models such as one proposed by Falmagne et al. (1975) entailed numerous unobservable parameters relating to various hypothetical memory states.

Contemporary sequential effects models emphasize probabilistic competition and cooperation between previous and oncoming stimuli. They integrate previous trial identities and continuously tune upcoming outputs over a given trial-run (e.g., Cho et al., 2002; Jones et al., 2013). These models explain sequential effects in largely cognitive terms, by positing short-term associative (learning) mechanisms that explicitly track and adapt to ongoing trial-events and presentation rates for the various trial-types. For instance, the Jones et al. (2013) framework includes a base-rate parameter that estimates the repetition rate, and an incremental learning parameter that adjusts the tradeoff between repetition and alternation for runs of two or more trials.

Nevertheless, there is little *a priori* motivation for an information processing system to guess upcoming trial identities, based on previous events with statistically equal long-run probabilities. Likewise, theorists struggle to explain the simultaneous importance of both the repetition and alternation patterns: Why is both repeating and reversing an action sometimes favored over everything in between? Why do repeating and alternating responses tradeoff in the face of very modest methodological changes, such as ITI timing?

This article proposes and tests a novel hypothesis that coupled oscillator dynamics explain sequential effects in response time. It is motivated by two recent discoveries; response time performances express a capacity for oscillatory entrainment and they also entail 1/ƒ scaling – a stochastic version of oscillatory synchronization (e.g., see, Kello et al., 2007; Amon et al., 2018). Since oscillatory dynamics also govern human locomotion, these reports imply that cognition and action are more closely intertwined than previously assumed.

By way of an overview, the Two-Alternative Choice Responses in Oscillatory Terms section reframes standard two-alternative forced-choice response time tasks and performances in terms of coupled oscillator dynamics. Trial-sequences indicating either “yes” (**R**ight-hand) or “no” (**L**eft-hand) responses are conceived as sequential signals that participants must track to correctly press the successive **R** and **L** response-buttons in two-choice decision tasks. That is, the role of successive choice stimuli is akin to a metronome in a conventional finger-tapping study, but the result of each choice determines which finger must be tapped on a given trial.

In this way, two-choice tasks are identified with a discrete version of the “HKB” or Haken et al. (1985) phase transition model of bimanual coordination. The discretized model is based on a sine circle map (deGuzman and Kelso, 1991). Each of 31 trial-histories, often targeted by the sequential-effects literature, is identified with a specific multifrequency polyrhythmic sequence typically targeted in the bimanual coordination and tapping literature (e.g., Schmidt et al., 1991; Kelso, 1995; Peper et al., 1995). The implication is that all trial-history permutations in a generic balanced experimental design, with equal affirmative right-hand (**R**) and negative left-hand (**L**) responses, equate to specific resonant modes of oscillatory entrainment. As such, coordinating one’s responses across trials is predicted to encourage various mode-locking oscillatory resonances to emerge in performance. Since successful responding requires mutual accommodation among both fingers used for responding, they are viewed as coupled oscillators. The relative speed and stability with which these resonances arise should correspond to the discrete HKB model’s dynamics. The Two-Alternative Choice Responses in Oscillatory Terms section ends by illustrating this core empirical prediction in a reanalysis of two classic sequential effects data sets.

In The Transition from Repetition to Alternation Dominance section, an additional sequential data set, published by Jones et al. (2013) is re-analyzed to test the oscillatory theory’s predictions about the apparent ITI dependent trade-off between the repetition and alternation effects noted by Kirby (1980). The oscillatory theory is generalized to explain sequential effects in error rates as well. Notably, the sections Statistical Analysis, Bifurcation Diagrams, and Devil’s Staircase overview the mathematical and statistical procedures used to work with the HKB model and the experimental data. Four additional studies are then presented to illustrate how oscillatory dynamics explain several commonly noted, and novel patterns in sequential effects. The section Persistence of Sequential Effects Under Cognitive Load explains how sequential effects behave under cognitive load. The section Controlling Dynamics With Characteristic Response Frequencies explores their sensitivity to the typical duration of response categories, which serve as a proxy for a characteristic oscillatory frequency. The section Distributed Cognition generalizes the core empirical patterns observed in the Section Persistence of Sequential Effects Under Cognitive Load to distributed cognition, in a dyadic decision task. The General Discussion section relates the findings to contemporary accounts of the dynamics of perception, cognition, and action, more generally.

## Two-Alternative Choice Responses in Oscillatory Terms

Responding in a conventional two-alternative forced-choice response time task is a fundamentally oscillatory activity, since responding requires repeated button-presses. In a choice task, one of each participant’s index fingers presses and releases a button on each trial. Each up-and-down motion constitutes a single oscillatory cycle. Thus, both the up-and-down motion of affirmative “yes” or target-present responses, and negative “no” or target-absent responses are oscillatory. From this perspective, a single response time approximates 1/2 the duration of a single button-press-release cycle.

Response times are measured from stimulus onset to the onset of a button-press. As such, a response time does not exclusively estimate the duration of a button-press cycle. For example, as when a task requires time-consuming, seconds-long judgments. However, for simpler tasks emphasizing perceptual identification, or predetermined responses to predetermined stimuli, the duration of a typical response time is much shorter, and more representative of a button-press cycle.

Forced-choice response tasks typically identify a participant’s dominant hand with targeted “yes” responses, and their non-dominant hand is identified with “no” or non-targeted responses. “Yes” responses tend to be faster than “no” responses, and dominant hands are more dexterous than non-dominant hands. Thus, for simplicity, this and all subsequent illustrations use an **R** to signify both “yes” and dominant right-hand responses. **L** signifies “no” and non-dominant left-hand responses since most participants are right-handed. The *crucial* implication is that **R** responses typically entail a “faster” oscillatory frequency than **L** responses.

In this framework, the successive identities of randomized sequences of presented stimuli serve as an ongoing driving signal that participants must mimic with oscillatory finger movements, as each stimulus, in turn, demands a response from one or the other finger. For instance, lexical decision requires participants to press a “yes” (**R**) key for legitimate English words and a “no” (**L**) key otherwise. If the stimuli presented on the first five trials are: *goat*, *glurp*, *frog*, *toast*, and *bave*, it corresponds to an **RLRRL** driving signal. That is, the stimulus sequence successively encourages or “drives” either an **R** or an **L** response on each trial (i.e., *word*, *nonword*, *word*, *word,* and *nonword*). Right-leading, or **R**-leading trial-histories are sequences that originate with an affirmative **R** response. This article emphasizes 31 **R**-leading trial-histories, from one to five-trials in length. They are the most widely studied and have the largest empirical record to draw upon. It is trivial to generalize this scheme to all possible permutations of **R** and **L** trials, including left-leading or **L**-leading trial-histories: **L**, **LR**, **LL**, and so on. However, all discussion of **L**-leading trial-histories is deferred until the letter rotation results section, below.

Finally, the two fingers used for responding are considered “coupled” by virtue of a shared mind, body, and nervous system. In action, locomotion, and even typing, successful coordinated limb activity requires continual mutual accommodation among the involved limbs (e.g., von Holst, 1939/1973; Bernstein, 1967/1996; Turvey and Carello, 1996). This is a minor conceptual leap; classic “finger waging” studies demonstrated bimanual coupling with horizontal finger movements (e.g., Kelso et al., 1981; Kay et al., 1987; Kelso, 1995), response time tasks use similar vertical finger movements. Next, the basics of a discrete, circle map version of the Haken et al. (1985) bimanual coordination model, oscillatory entrainment, and mode-locking Farey trees are discussed. Readers familiar with these topics may wish to skip ahead to The Transition from Repetition to Alternation Dominance section.

### A Phase-Attractive Circle Map

An oscillatory characterization of button-press responding motivates the adoption of a discrete circle map difference equation as a model for the relatively consistent empirical patterns reported in the sequential dependency literature. A circle map is a difference equation used to characterize frequency entrainment patterns in coupled oscillators (e.g., see Bak et al., 1985; Bak, 1986; Halsey et al., 1986; Schmidt et al., 1991; Shroeder, 1991; Treffner and Turvey, 1993). Generic circle maps are commonly adapted for specific mathematical or physical applications. The circle map depicted in Equation 1 is a discretized version of the ordinary differential equation entailed in the HKB model of bimanual coordination (deGuzman and Kelso, 1991):

(1) $$\phi_{n+1}=\phi_{n}+\Omega -\frac{K}{2\pi }\left[1+\mathrm{Acos}\left(2\pi \phi_{n}\right)\right]\sin\left(2\pi \phi_{n}\right),\mathrm{modulo}\;1$$

Equation 1 is a *phase attractive* difference equation. It captures the in-phase and antiphase relations intrinsic to bimanual coordination. Relative phase, ϕ, is normalized to the unit interval, and refers to discrepancies in two oscillator’s phases, so for instance, a repeated result where ϕ=0 signifies two in-phase, equal-frequency oscillators. Omega (**Ω**) is the driving input or “biasing” signal. The coupling strength between the oscillators is **K**, and **A** refers to the relative prominence of the antiphase attractor. An extensive program of research in human locomotion revealed two fundamentally preferred, or phase *attractive* relations in the coordinative dynamics of action (e.g., see Kugler and Turvey, 1987; Schmidt et al., 1991; Kelso, 1995; Turvey, 2007). In bimanual coordination, the dominant attractor is in-phase, as when one taps two separate fingers in unison to a single beat. Since both fingers tap and rebound together, relative to each other, they share identical locations in their phase spaces. This is known as an *in-phase* relative phase relation.

*Antiphase* coordination is illustrated in walking. While walking, each leg takes turns lifting and swinging. At any given point in time, one’s legs tend to be in *opposing* positions in their respective oscillatory cycles – their typical relative phase relationship is approximately antiphase. Of course, with practice, one can learn or follow more complex phase relations (e.g., skipping), but the *in-phase* and *antiphase* relative coordination patterns are intrinsic to human physiology and action (e.g., Kelso, 1995; Peper et al., 1995). The remainder of this section is dedicated to unpacking the relevant parametric influences and dynamics of Equation 1. Later sections further elaborate these themes in the context of studies and related figures.

### Farey Series: Choice Stimuli in Oscillatory Terms

**Figure 1:**
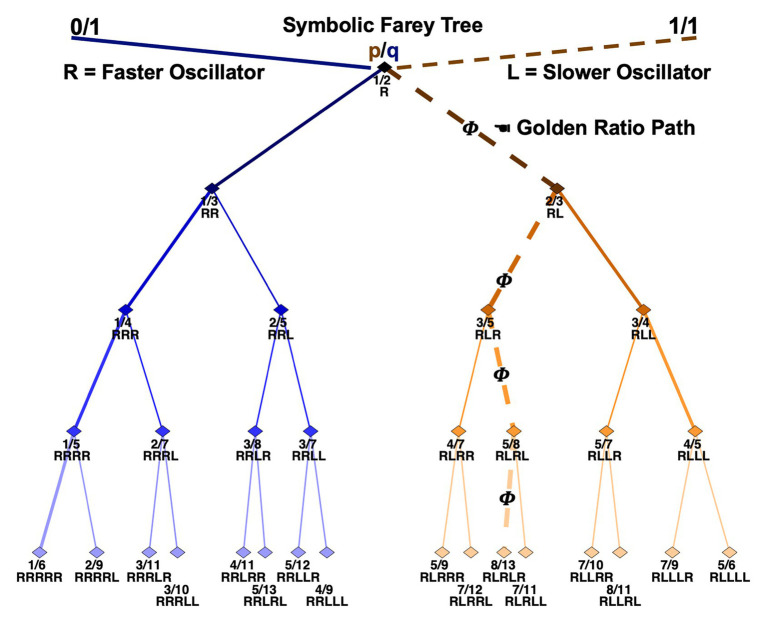
An illustration of the Farey tree branching relationships capturing all 31 permutations of **R**-leading trial-histories. The branching relationships also depict the mode-locking attractors of generic pairs of coupled oscillators with distinct individual frequencies. The labels terminating each branch indicate the mode-locking ratio in the form of a mediant fraction, and as a specific trial-history sequence. Five levels of trial-history sequences are displayed. The first trial of the trial-histories is always **R**, and progressively more recent trials are listed in succession. Thus, the first trial in the sequence is the oldest trial, and the trial terminating a sequence is the most recent trial. Notably, the **R**s and **L**s in popular accounts of the Farey tree are typically reversed, relative to these, indicating paths based on branching to the left or right on the diagram itself. All the mathematics are preserved with the exchange of the letters used here to code sequences of **R** and **L** finger movements.

A Farey tree is a hierarchical number-theoretic construction that depicts the phase attractive frequency ratios a pair of physically or informationally coupled oscillators will tend to occupy. Figure 1 illustrates a mode-locking Farey tree. The top-level depicts the seed ratios 0/1 and 1/1. An oscillator with a higher natural frequency is signified on the 0/1 side of the tree. An oscillator with a lower natural frequency indicated by the 1/1 side of the tree. A matrix-encoded Stern-Brocot tree explains the origins of the seeds.^1^

On each successive tree level, new fractions are computed by separately summing the numerators and denominators of the two *nearest* adjacent ratios on any preceding level. Each fraction represents a potential coupling ratio in which a higher frequency oscillator will tend to entrain with a lower frequency oscillator. For example, summing 0/1 and 1/1 on level zero yields 1/2 on level one. Physically, this indicates the higher frequency oscillator will complete two cycles or pulses along with a single cycle of the lower frequency oscillator, and so on down the tree. Continuing, the *mediant* sum of 0/1 and 1/2, is 1/3 and 1/2 and 1/1 is 2/3; these integer ratios depict 1:3 and 2:3 cycle entrainments, and so on down the levels (Schmidt et al., 1991; Kelso, 1995). Thus, each ratio on the tree indicates a preferred resonant mode for two coupled oscillators.

This article tests a working hypothesis that tracking stimulus-driven button-press sequences, using fingers with slightly discrepant response times, as they track various Farey tree paths explains the basis of sequential effects. Every permutation of **R**-leading trial-histories is identified with a rational fraction on the Farey tree. This amounts to a symbolic depiction of the branching paths needed to arrive at each Farey tree entrainment ratio. For instance, the path to 2/7 begins at 1/2, then 1/3, then 1/4, then 2/7 or from **R** to **RR** to **RRR** to **RRRL**. A common point of confusion arises in translating continuous, analog oscillatory orbits, indicated by the exact ratios, into the symbolic Farey tree paths, indicated by sequences of **R**s and **L**s. The letter positions indicate place values analogous to numeral locations in decimal numbers, or letters in a word (Peitgen et al., 1992/2006). Both their continued fraction and decimal equivalents make this clear; each step down the tree takes a smaller “bite” from the unit interval, as two paths diverge. Strictly speaking, the symbolic addresses reference the ever-shrinking intervals *between* their parent ratios, while the ratios themselves reference points at the center of these intervals. One implication is that from the perspective of the exact fractional ratios, the symbolic paths always entail an implicit or “unvoiced” terminating 1:1 “beat.” For example, the 1/2 ratio literally indicates one **L** response for every two **R** responses, something like **R**-(**R** and **L**), but is denoted simply as **R** on the tree. The omission is ideal for forced-choice tasks since simultaneous (in-phase) **R** and **L** responses are always forbidden.

The symbolic addresses can be verified arithmetically in several ways, as by the method of continued fractions (see Kappraff, 2002). One verification route, used extensively in the error-rate analyses below, is to first rank the ratios in *ascending* order by level, and then in *descending* order by magnitude within each level (e.g., [1/2], [2/3, 1/3], [3/4, 3/5, 2/5, 1/4]…). Next, compute the binary representation of each ratio’s numerical rank. This returns the fraction’s address in terms of ones and zeros. For instance, 2/7 ranks 14th in a level by magnitude sort, and the binary representation of 14 is: **1110**, or **RRRL** in this illustration. The golden ratio, 8/13, ranks 21st, and the binary representation of 21 is: **10101**, or **RLRLR**, the *alternation* pattern noted earlier. This binary ranking is formally identified with the continued fraction expansion of each Farey ratio (Schroeder, 1991; Kappraff, 2002). In this way, all 31 permutations of one- to five-trial histories typically studied in sequential effects analyses are formally isomorphic to a description in terms of potential oscillatory entrainments.

What is the basis of these preferred integer-ratio mode-locking resonances? Well, every child who’s used a swing implicitly discovers that oscillators get the most potent resonant “kick” when they are driven at regular and specific points in their cycle, or phase space (Schroeder, 1991). This explains the *phase attractive* status of the integer ratios depicted by the Farey tree. Importantly, the upper-level ratios with smaller denominators yield more stable entrainments than the lower-level ratios with larger denominators (Treffner and Turvey, 1993).

#### The Devil’s Staircase

**Figure 2:**
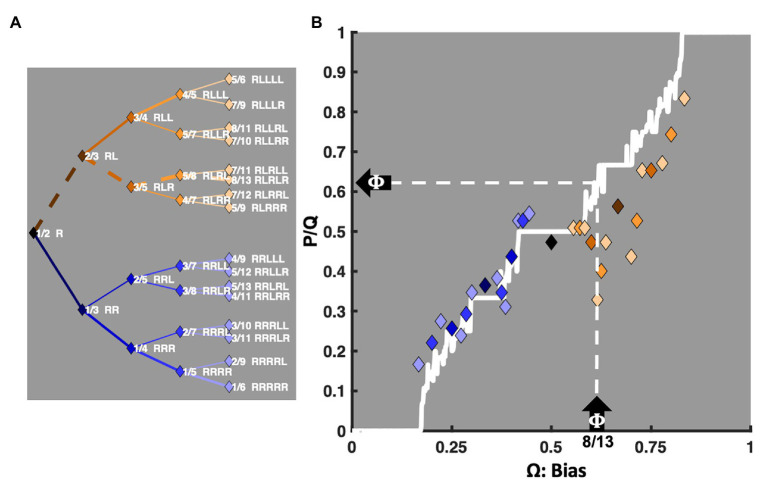
An illustration of the relationship between the Farey tree ratios and the Devil’s staircase. The *X*-axis of plot (B), labeled **Ω**, depicts the biasing or driving inputs to the bimanual model. For example, consider the upward pointing arrow on the *X*-axis indicating 8/13 (0.618), the golden ratio (**Φ**), as a driving frequency. The left-pointing arrow on the *Y*-axis then indicates **Φ** as the likely Farey ratio (p/q) at which two suitably coupled oscillators, such as those entailed by the bimanual model, will display mode-locking. The corresponding mediant ratios are indicated on the left, in plot (A). All 31 trial-history permutations are formatted sideways as a Farey tree, and approximate their respective locations on plot (B)’s *Y*-axis. The solid white line bisecting plot (B) is the Devil’s staircase. Each plateau in the staircase indicates the width of a corresponding Arnold tongue, an attractor basin in which a range of biasing frequencies will likely yield a single mode-locking ratio. The various plateau widths result from the fact that ratios with smaller denominators are more stable than ratios with larger denominators. Notably, the staircase was generated with **A** = 0.85, and **K** = 0.95, and driven from **Ω** = 0 to **Ω** = 1 in increments of 0.001. For simplicity, the noted **A** and **K** parameters were fixed for this and all subsequent plots and statistical contrasts in this article. The mean response times for Remington’s (1969) data set, taken from a table of condition means appearing on Luce (1986, p. 288), were normalized to the unit interval, and plotted against the staircase. Each diamond marker indicates a specific trial-history in terms of the color of its corresponding Farey tree branch on plot (B). The correlation between the normalized response times and their predicted Farey ratios, encoded by the Devil’s staircase is *r*(29) = 0.89, *p* < 0.0001. Notable exceptions are the ratios on the golden ratio path. They are related to antiphase bimanual coordination and discussed in The Transition From Repetition to Alternation Dominance section.

The coupling strength between two oscillators determines their relative stability in the various potential entrainment ratios depicted on the Farey tree. A function, often called the Devil’s staircase, relates the characteristic frequency ratio (**Ω**) used to *drive* a pair of coupled oscillators and their expected Farey ratio. The diagonal stepped white line bisecting Figure 2B illustrates this function. For a given **A** and **K**, the length of each staircase plateau indicates the width of each mode-locking region, called an Arnold tongue (deGuzman and Kelso, 1991; Peper et al., 1995). The plot’s *X*-axis depicts the input frequency of the driving force, denoted as **Ω**. Its *Y*-axis depicts the most likely or *expected* resonant Farey ratio, p/q, in units of average rotations per iteration. In this framework, **Ω** indicates the ratio corresponding to the trial-sequence to which a participant must respond. To the extent the participant’s performance is mostly accurate, **Ω** is understood as a driving input, biasing the dynamics observed in performance. Likewise, p/q measures the relative amount of time required to execute each trial-sequence, from fastest to slowest. That is, p/q predicts how quickly the various mode-locking ratios can be executed. The widths of the staircase “steps” indicate the widths, along the **Ω**-axis, of each Farey ratio’s attractor basin. Figure 2B depicts Remington’s (1969) trial-history means, normalized to the unit interval, and colored in terms of their referent Farey ratio. They are reliably correlated with their predicted locations on the Devil’s staircase, *F*(1, 29) = 120.03, *r^2^* = 0.80, *p* < 0.0001. The relationship is quite good, especially given the golden ratio’s potential to deviate from the staircase, discussed in the section A Preliminary Assessment: Classic Sequential Effect Data Sets, below. Going forward, the staircase serves as the primary predictor in correlations with the relative durations of unit-normalized response times for the 31 trial-history sequences.

##### Parameter Selection

As noted, the staircase plateaus indicate the dominant resonant mode for a generic pair of coupled oscillators, given a particular driving frequency ratio (**Ω**) and coupling strength (**K**). However, the bimanual coordination model is more complex, as it entails its own intrinsic in-phase and antiphase attractors. The relative importance of the in-phase and antiphase attractors is controlled by a third parameter (**A**). Notably, both dominant 1:1 phase relations are unavailable in two-choice tasks. Pressing two buttons at once is forbidden and participants rarely, if ever, execute superfluous response movements, such as simultaneously pressing one button while lifting the other finger.

Instead, speeded button-press responding likely requires symmetry-breaks, inhibiting one hand. Nevertheless, the 1:1 antiphase attractor is less stable at both higher oscillatory frequencies, and with increasing discrepancies between two oscillator’s frequencies (Kelso, 1995). Response times result from speeded acts, and the extant sequential effects literature indicates alternation effects, which resemble antiphase coordination, are more common for ITI’s >500ms. Likewise, bimanual coordination reports suggest the critical frequency for a phase transition from antiphase to in-phase coordination is just below 2.2Hz or about 450ms (deGuzman and Kelso, 1991). Therefore, we set the model’s **A** parameter to **A** < 1, wherein only the in-phase attractor is stable, but close to the threshold where antiphase coordination stabilizes as well: **A** = 0.85. Importantly, that the antiphase attractor is *unstable* does not mean it is *unavailable*. In fact, there are several circumstances in which performance relating to antiphase coordination is expected to be favored in the following studies.

The core hypothesis that sequential effects emerge as a consequence of coupled oscillator dynamics implies a need for a strong coupling, and well-articulated plateaus in the Devil’s staircase. The mode-locking phase relations are largely unconstrained under weak coupling, which undermines the uniqueness of the model’s predictions in that situation. The relative phase surface has many local minima, that tend to obviate selection *via* optimization. As such, **K** = 0.95 was set in conjunction with **A** = 0.85 to produce wide Arnold tongues and a connected staircase. An initial examination of Remington’s (1969) data indicated these choices were reasonable (e.g., Figure 2B). Thus, for any triad of parameters (**Ω**, **K**, **A**), the model’s staircase provides comprehensive response time predictions for all **R**-leading one- to five-trial histories.

Together, the **A** = 0.85 and **K** = 0.95 parameters yield chaotic dynamics in the *model* (see deGuzman and Kelso, 1991). Some evidence suggests forced-choice performances express chaotic dynamics (Kelly et al., 2001). However, this question is set aside here because decisively demonstrating chaotic dynamics in *performance* is methodologically and statistically challenging (Mitra et al., 1997). Notably, informal exploration of nearby parameter sets indicated that modest changes had little qualitative impact on the macro-features of the staircase, in Figure 2B, that are essential for testing predictions (e.g., **A** and **K** ±0.05). In addition, non-chaotic parameter sets did not satisfy the core requirement that bistability be nearby, in the context of well-articulated staircase plateaus (e.g., **A** = 0, **K** = 0.95).

### Oscillatory Entrainment Predictions

In addition to the core mode-locking hypothesis, additional qualitative predictions relating to the basis of sequential effects can be derived. *Self-resonance* likely explains the dominance of the repetition effect for faster ITI tasks. Fingers are only a few inches in length, relatively light, and thus have short natural periods. If a task’s pace is just right, a single finger can capitalize on the button-spring to maintain and speed the period of successive button presses. This form of self-resonance is akin to a child bouncing on a pogo stick. For maximum bounce, the downward forces generated by a child’s jumping motion are in balance with the upward force of the spring’s potential energy. If the child is a bit too light or heavy, repeated bouncing becomes more effortful.

As the pace of a decision task slows below a finger’s natural period, it will tend to erode the dominance of the repetition effect. Thus, self-resonance may explain why the **RRRRR** pattern is typically faster in perceptual tasks with ITIs less than about 500ms. In addition, it predicts that some self-resonance in the slower left-hand **RLLLL** pattern could emerge, and at least overtake its nearby Farey tree neighbors. However, in conventional tasks **RLLLL,** is unlikely to overtake the **RRRRR** pattern since the **RLLLL** pattern concerns a slower oscillator, the non-dominant hand.

The intrinsic antiphase bimanual coordination attractor likely explains reports of crossovers from the relative dominance of repetition to alternation in tasks with slower ITIs, or experimental preparations that otherwise hamper the potential for self-resonance to emerge. This is because antiphase coordination is intrinsic to bimanual coordination but stabilizes at lower frequencies. In the absence of a strong potential to support self-resonance, the antiphase pattern may outpace its nearby Farey tree neighbors, and even repetition.

#### A Preliminary Assessment: Classic Sequential Effect Data Sets

**Figure 3:**
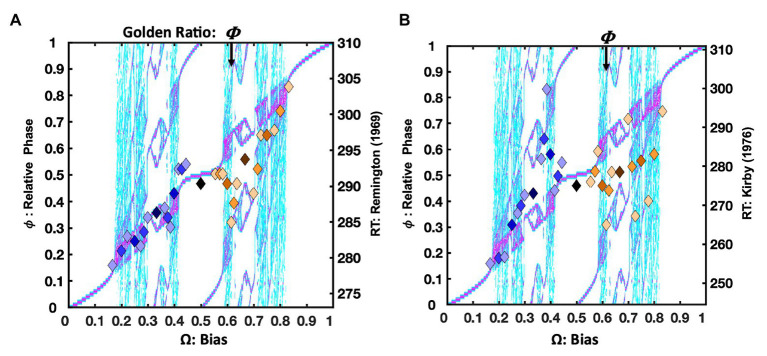
For continuity, plot (A) again depicts the Remington’s (1969) data as color-coded diamond markers, depicting the 31 ratios, just as in Figure 2B, now atop a detailed bifurcation diagram portraying the potential range of the relative phase relations (ϕ) that arise in the model’s dynamics. Rather than the whole-orbit counts of p/q, relative phase (ϕ) relates two oscillators as an angular difference in their respective cycles. Regions in white indicate the absence of a phase attractive region, turquoise shades (lighter gray) indicate relatively weaker phase attractive regions, and pink shades (darker gray) indicate stronger phase attractive regions. The bifurcation diagram illustrates how the attractors encoded by the staircase can be achieved through a variety of relative phase relations. The Devil’s staircase is an asymptotic summary of these dynamics, in integer counts of both oscillator’s respective cycles. Plot (B) depicts (Kirby’s, 1976) delayed ITI version of a two-button task. The raw data was unavailable. It was estimated from measurements of an enlarged photograph of a plot in the original published article and transformed to the millisecond scale, based on the graph’s axes. The correlation between the normalized latencies and their predicted Farey ratios is weaker than Remington’s data, *r*(29) = 0.52, *p* < 0.003. While the markers deviate more from the staircase than the Remington data set, the bifurcation diagram indicates the deviations are generally consistent with the model’s phase attractive topology, as indicated in the bifurcation diagram. Both right-hand *Y*-axes in this and all subsequent response time plots depict the trial-histories’ untransformed latencies in milliseconds (ms).

An assessment of these core oscillatory entrainment predictions begins by revisiting Remington’s (1969) sequential effects data. The response time data appear in Figure 3A (Remington, 1969). The *X*-axes in Figure 3 portray each trial-history as a driving signal, in decimal equivalents to its mediant fraction (e.g., **RRRL** = 2/7 = 0.286). Again, the diamond markers are color-coded according to their Farey tree branching, now plotted over a detailed bifurcation diagram of the model’s relative phase space (ϕ) along the unit interval. The normalized response times approximate relative phases, on the *Y*-axis, since they indicate the relative amount of time required to execute the sequences. For reference, the right-hand *Y*-axes indicate the corresponding raw mean response times in milliseconds.

As previously noted, the association between the Remington means and the staircase is strong. Given a driving frequency **Ω**, the staircase depicts the expected entrainment ratio – the attractor. However, circle maps and the bimanual model entail subordinate phase-attractive regions as well. Their locations and relative strengths are a function of the coupling parameters, **K** and **A**. Additional phase attractive regions indicate the availability of alternative coordinative regimes, such as periodic, quasiperiodic, chaotic, and limit cycles. Intrinsic dynamical variability, or phase drift induced by noise make these alternative relative phases available.

Figures 3A,B bifurcation diagrams depict the relative strength of the phase attractive regions for the bimanual coordination model where **K** = 0.95 and **A** = 0.85. The notable staircase exceptions for the Remington (1969) data set are fractions in the Fibonacci sequence approaching the golden ratio: **RL**, **RLR**, **RLRL**, and **RLRLR**. Past 2/3, the mean response time for the Remington data decreases with each successive alternation. In this region, the coordination model’s bifurcation diagram reveals the availability of alternative, higher frequency, relative phase relations that fall well below the staircase expectation. In a generic circle map, the golden ratio is among the *least* stable ratios (Treffner and Turvey, 1993). However, in this context, it is phase-attractive, as antiphase coordination is an intrinsic attractor in the bimanual coordination model.

Figure 3B illustrates data from Kirby’s (1976) *delayed* ITI version of the task, after significant on-task practice. Apparently, the slower task pace erodes the self-resonance advantage for several of the smaller ratios, ranging from 1/4 to 2/5. They trend above the phase-attractive diagonal, despite each ratio emphasizing the dominant right finger. Similarly, the long ITI tends to erode the relative disadvantage of the larger ratios. Again, the notable exception being the ratios in the Fibonacci sequence approaching the golden ratio.

Participants (and organisms) commonly behave *as if* they were optimizing, influencing, or accommodating parameters controlling an activity’s mathematical formulation. Classic examples include time-to-contact (Lee, 2009), juggling (Beek and Turvey, 1992), catching a baseball (Oudejans et al., 1996), hula-hooping (Balasubramaniam and Turvey, 2004), Schmidt’s, Fitts’s, and Hick’s law (Hick, 1952; Flach et al., 1996; Wijnants et al., 2009) and of course, bimanual coordination (e.g., Haken et al., 1985; Kugler and Turvey, 1987; Kelso, 1995; Peper et al., 1995). All the aforementioned activities can be described mathematically, and their performance profiles relate to coordinative relationships entailed in those descriptions (Turvey, 2018). Going forward, the goal of the studies and analyses is to test the working hypothesis that sequential effects can be understood and controlled with manipulations relating to oscillatory principles and parameters.

## The Transition From Repetition To Alternation Dominance

The repetition pattern tends to dominate in tasks that use relatively short ITIs. By contrast, alternation tends to dominate in tasks that use longer ITIs. Kirby’s (1980) review is largely responsible for marshaling evidence for these competing tendencies. Historically, the faster time-course of the repetition effect suggested automatic facilitation, such as a quickly decaying memory trace. The slower time-course of the alternation effect suggested a performance strategy. However, these basic tendencies do not present as hard-and-fast rules. Example exceptions include Kirby’s (1976) studies in which participants were asked to *anticipate* either repetition or alternation prior to completing experimental sessions. In each condition, the anticipated pattern yielded faster performance. The primary caveat was that anticipation was more effective in tasks with ITIs >1s. Also, the Remington (1969) task, noted earlier, used a 1s ITI. While the repetition pattern is fastest in that study, the alternation pattern is faster than nearby trial-history ratios.

Next, in the section A Contemporary Sequential Dependency Data Set, the Jones et al. (2013) data are analyzed and depicted in terms of the oscillatory framework. The goal is to illustrate how the Jones et al. (2013) trial-history bias manipulation can be interpreted in oscillatory terms. Following that, data from two speeded and one standard “Hick’s Law” experiments are analyzed to compare the impact of the Jones et al. (2013) learning-based manipulation and the speed-accuracy trade-off entailed in the Hick (1952) style response time studies.

### A Contemporary Sequential Dependency Data Set

Jones et al. (2013) tested a hypothesis that Bayesian learning explains sequential dependencies. They introduced a potent manipulation of the presentation rate of repetition and alternation trials to over-present either repetition or alternation in the presented trials. However, successfully influencing performance with instructional and presentation-rate manipulations does not preclude a formative role for dynamics as a basis for these performances.

The oscillatory hypothesis implicates entrainment dynamics as foundations for controlling performances that may subsequently be leveraged *via* intentions and experience (e.g., instructions and learning) to accomplish and maintain goal-directed activities (Van Orden and Holden, 2002; Amon et al., 2018). Thus, entrainment predicts that oscillatory factors may spur the kinds of changes that are eventually reflected and amplified in learning studies, for instance (e.g., Mitra et al., 1998). As the present analyses demonstrate, the trial presentation-rate manipulations influenced the response times and error rates for the trial histories and their respective locations on the Devil’s staircase and Farey tree.

#### Method

##### Participants and Procedure

Each of the 28 Jones et al. (2013) participants completed two experimental sessions consisting of 3,722 trials across 32 trial-blocks. The data were made available as a supplement to the published article. In each session, either the repetition or alternation pattern was overrepresented from the base recurrence rate expected in sequences governed by standard trial-randomization procedures.

On each trial, participants pressed one button if a small white dot was presented above a fixation point, and another button if it was presented below the fixation point. Condition order and the **R** vs. **L** response-button mappings were counterbalanced. The base **R** and **L** response rates were equal, but the sequences were controlled so that 2/3 of the trials targeted for analysis were repetition or alternation trials, respectively. The Jones et al. (2013) design also included unanalyzed filler trials, and 10 buffer trials at the beginning of each block. The initial 10 buffer trials in each participant’s first block were eliminated from this analysis, and the remaining 3,712 trials were analyzed as a single contiguous block. For consistency with previous and subsequent analyses presented in this article, all analyses, including the present Jones et al. (2013) analyses were computed to a five-trial depth. Preliminary analyses revealed an absence of qualitative counterbalancing differences; all our subsequent analyses collapsed across that factor. Readers are referred to Jones et al. (2013) article for complete methodological details.

##### Statistical Analysis

Custom software, coded in MATLAB (2020), searched for all the targeted trial-history sequences. It used each participant’s first valid trial as the origin for sequence identification. Every subsequent observation also served as a potential sequence origin. As an example, if the first six trials were correct, and happened to be **RRRRRR**, the following sequences would result: two **RRRRR**, three **RRRR**, four **RRR**, five **RR**, and six **R**. This is because each participant’s data was rescanned to search and classify each of the 31 trial-histories separately. If the first or last **R** in the above example was an error, the correct trial-history counts for each trial-type would be reduced by one. The lexical decision and rotation tasks, below, used DirectRT^®^ and Superlab^®^, respectively, for data collection.

Mean response times were computed only for the targeted sequences in which *all* response times comprising the sequence were correct, and within a 200–980ms censorship boundary (the censorship boundaries of the remaining studies are noted in their respective results sections). Once the means for all permutations of a participant’s trial histories were computed, they were normalized to the unit interval. This normalization was done by subtracting the fastest of their 31 means from each trial-history mean and dividing the result by the range of the 31 means: (${\bar{X}}_{i}$ − Min)/(Max − Min). Averages for the trial-histories were then taken across participants. The resulting averages were then plotted in figures and used for subsequent statistical analyses. Since the Devil’s staircase predicts the relative latencies of the trial-histories, its p/q value at the **Ω** corresponding to each trial-history ratio was used as the predictor in correlations with the unit-normalized means. The correlations were transformed to coefficients of determination (*r^2^*) and *F*-ratios using formulas available in standard references (e.g., Cohen et al., 2013).

Since responses could appear in more than one sequence of a given type, error counts for each trial history were computed as the number of *unique* errors divided by the total number of individual responses captured as members of a given sequence type. As an example, for Participant 28 in the Jones et al. (2013) repetition data set, the analysis identified 925 observations comprising 185 **RLLLL** sequences (5 × 185 = 925). Sixty-seven of the 185 sequences contained errors. However, only 56 errors were unique, and the error rate was thus computed as 56 ÷ 925 = 0.0605. Mean response times were computed using the responses from the remaining 76 error-free **RLLLL** sequences that fell inside the 200–980ms censorship boundary. Finally, the participant’s trial-history means were normalized to the unit interval, and then averages for each of the 31 trial histories were computed across all 28 participants.

All error rates for Farey tree analyses are reported as proportions, and coefficients of determination and *F*-ratios were computed using Spearman rank-order correlations between a binary ranking of the Farey tree ratios and a ranking of the corresponding error rates. Ordering the ratios in terms of their binary rank, discussed earlier, offers a detailed account of the relationship between the error rates and the Farey tree’s shape. The binary ordering corresponds to sorting each successive level of the tree from the largest to the smallest ratio (e.g., [1/2], [2/3, 1/3], [3/4, 3/5, 2/5, 1/4]…). Likewise, the error rates corresponding to the subset of ratios within each level are sorted from largest to smallest. Then a Spearman rank-order correlation is computed. With this ranking, a Spearman rank-order correlation of +1 results when all the error rate magnitudes (largest to smallest) within each tree level equate with the binary rank of the ratio magnitudes (largest to smallest) within the same Farey tree level. Notably, an additional *DF* was subtracted from all Spearman *F*-tests since the error-rate rank of the root mode-locking ratio (1/2) cannot vary, as there is only one entry.

##### Bifurcation Diagrams

The circle map bifurcation diagrams were generated from 1,000 evenly spaced values of **Ω** on the unit interval. For each value, Equation 1 was seeded with a unit-interval random number and iterated 10,000 times, with **A** = 0.85 and **K** = 0.95. The values from final 100 iterations of each **Ω** were then depicted on a contour surface.

##### Devil’s Staircase

The staircase was generated iteratively, just as the bifurcation diagrams. However, the *modulus 1* operator was disabled (see Peper et al., 1995) and 2,500 of the resulting values for each **Ω** were averaged to estimate p/q.

#### Results and Discussion

##### Repetition

**Figure 4:**
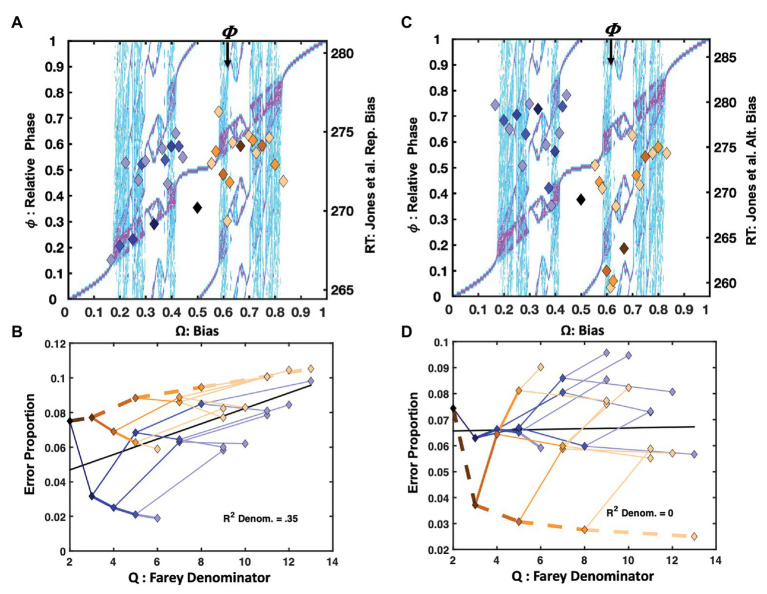
Plot (A) depicts unit-normalized response times for the (Jones et al. 2013) condition emphasizing the repetition pattern as color-coded markers, indicating the 31 Farey ratios, over the relative-phase bifurcation diagram. The *X*-axis tracks the input bias (**Ω**), the left-hand *Y*-axis indicates the resulting relative phases (ϕ). Again, the range of the untransformed mean response times is on right-hand *Y*-axis in ms. The trial-histories are generally consistent with the predictions of the bimanual model. Correlations indicate the left-side ratios approximate the staircase and deviations tend to occupy phase attractive regions of the bifurcation diagram. The primary exceptions to the staircase on the plots right side are the golden ratio and the **RLLLL** patterns. The normalized response times for the alternation condition appear in plot (C). Trial histories on the golden ration path show a clear advantage, and the underrepresented repetition ratio (**RRRRR**), and its neighbors on the plot’s left are notably slower. Plots (B,D) depict the error rates in terms of a sideways Farey tree; as in Figure 2A, the root ratio of 1/2 is on the left, rather than at the top of the plot. The error rates for each of the 31 trial histories are again plotted as a function of their color and branching relationships. To the extent the color-coded branching tracks the qualitative sideways tree-structure, as depicted in Figure 2A, the error-rates track the relative stability of the Farey ratios. In plot (B), the repetition pattern has the lowest error rates, and is most stable. In plot (D), the golden ration path has a clear stability advantage. Error-rates for ratios deviating from the path are comparable to the remaining ratios. This suggests the learning resulting from the trial-history bias manipulation does not generalize to closely related ratios.

The Jones et al. (2013) repetition condition presented **RRRRRR**, **LLLLLL**, and all their precursors on 2/3 of the trials. However, only the 31 trial-histories noted in Figure 1 are considered in this analysis. In Figure 4A, the mean response times for each trial-history in the Jones et al. (2013) repetition bias condition are depicted as points in a scatter plot over the bimanual model’s relative phase topology.

Overall, the **RRRRR** repetition pattern produced the fastest response times and the lowest error rates. The trial histories on the plot’s right are compressed relative to the staircase. The temporal compression on the plot’s right likely arose since the Jones et al. (2013) protocol increased repetition rates for both the right and left hand, yielding a slight practice advantage for closely related sequences. For example, the **RLLLL** pattern is among the fastest ratios for **Ω** > 0.5. Despite the alternation ratio’s underrepresentation, on only 4% of trials, it yielded a response time advantage relative to neighboring ratios. The bimanual antiphase attractor likely facilitated this advantage. The correlation between the trial histories and the Devil’s staircase prediction was reliable, *F*(1, 29) = 9.14, *r^2^* = 0.24, *p* = 0.005.

The location of the ratios on the Farey tree indicates the relative stability of each ratio. Ratios with larger denominators are less stable and should garner more errors than ratios with smaller denominators. Likewise, ratios emphasizing the more stable, higher-frequency, and affirmative **R** responses are less error-prone. The repetition condition error rates for each of the 31 trial-histories are plotted (sideways) in Figure 4B, in terms of their denominators, and branching on the Farey tree. The denominators of the trial-history ratios are plotted on the *X*-axis, and the error-proportions for the corresponding ratio appear on the *Y*-axis. The ratios corresponding to the **RRRRR** and **RLLLL** paths were more accurate than an expectation given by unbiased trial-history probabilities. In addition, the golden ratio path and alternation ratio produced higher error rates than their Farey-tree based expectation.

An analysis of the error rates indicates relatively strong consistency with the Farey tree, with the exception of the experimentally biased ratios. The Spearman rank correlation for the repetition condition was *F*(1, 28) = 145.58, *r^2^* = 0.84, *p* < 0.001. Thus, the remainder of the errors to ratios were generally consistent with the essential prediction that ratios corresponding to less stable mode-locking are more error-prone. Despite the few noted error-rate exceptions related to the sequence bias manipulation, the overall association between the denominators of the ratios, and the error rates was also reliable, *F*(1, 29) = 15.62, *r^2^* = 0.35, *p* < 0.001. This relationship is generally predicted to be positive since larger denominators correspond to less stable mode-locking. The relationship is depicted by a black line on this and all subsequent Farey tree error-rate plots (e.g., see Figures 4B,D).

##### Alternation

The alternation bias yielded a strong response time advantage for **RLRLR**, and its precursors on the golden ratio path. The alternation condition response times appear in Figure 4C. In this case, the ratios on the left side of the relative phase plot were slower, with the underrepresented repetition pattern being among the slowest. The manipulation was potent enough to produce a *negative* association between the staircase and response times *F*(1, 28) = 5.18, *r^2^* = 0.15, *p* = 0.03.

The alternation emphasis was sufficient to perturb the general relationship between the ratio denominators and error rates, effectively eliminating the correlation observed in the repetition condition, as indicated by the horizontal black line in Figure 4D. Overall, the pattern of errors echoed the response time pattern. Sequences on the golden ratio path were advantaged relative to nearly all other trial histories. Nevertheless, the error rate correspondence with the Farey tree in the alternation condition was reliable but weaker than in the repetition condition, *F*(1, 28) = 61.34, *r^2^* = 0.69, *p* < 0.001.

##### Summary

The Jones et al. (2013) repetition condition yielded faster response times for the two ratios, and their precursors, that were largely comprised of repeats: **RRRRR** and **RLLLL**. Despite being strongly underrepresented, the alternation pattern echoed the modest response time advantage noted earlier, in the Remington (1969) and Kirby (1976) data sets. The error rates tracked both the magnitude of the mode-locking ratio’s denominators, and the Farey tree’s overall shape. The alternation manipulation yielded the expected advantage for sequences on the golden ratio path. However, the remaining patterns, including repetition, failed to display an advantage in response time when the trial histories emphasized alternation. Thus, overrepresenting the recurrence rates of specific patterns yields learning, relative to underrepresented patterns (Jones et al., 2013).

The learning, however, was highly specific, and did not generalize to unpracticed trial histories. In the repetition condition, as soon as the trial histories deviated from the repetition path, error rates dramatically increased. The same pattern emerged for the alternation condition. The pace of the Jones task was rapid, stimuli were displayed for 60ms, and trials timed after 1s. Despite the low repetition rates in the alternation condition, repetition trials managed to maintain lower error rates in the alternation condition, compared to alternation error rates in the repetition condition. Since the **R**-leading repetition pattern relies on self-resonance with response buttons, under-presenting it was not too disruptive. Likewise, when alternation was underrepresented, it too was favored relative to neighboring ratios – both patterns are consistent with oscillatory dynamics and the bimanual model.

The oscillatory framework predicts that changes to the dynamics of the coupling between participant and task will give rise to similar trade-offs between repetition dominance and alternation dominance. Next, we describe a simple reaction time task modeled after the classic Hick’s Law reaction time paradigm.

### Fixed and Rapid vs. Slower and Variable Trial Pacing

Oscillatory entrainment principles point to a more direct way to induce a transition from repetition to alternation dominance in a perceptual identification task. This study seeks to achieve the same basic control over the transition from repetition to alternation dominance, noted in the Jones et al. (2013) study, by manipulating the nature of dynamic coupling between participant and task.

The task is modeled on classic Hick (1952) protocol but adapted for a PC and keyboard. At the beginning of each trial, two square wire-frame outlines were presented in black against a white background, as a fixation stimulus. The squares were presented at the center of the display and spaced to align with the F and J keys, where participants placed their left and right index fingers to respond. The response signal grayed-in the targeted square above the targeted finger and response button. The untargeted square remained unfilled.

In this study, the manipulations emphasize trial pacing and stability. The standard version of the task uses slow and variable ITIs that impede self-resonance. This will tend to advantage the golden ratio path, as it is favored on slower timescales, and intrinsic to bimanual coordination. The two remaining conditions impose regular, tempo-based response deadlines, that are paced to be 100 and 200ms *faster* than each participant’s average response time in the standard version of the task. The faster pace is expected to favor self-resonance.

#### Method

##### Participants

Eight undergraduate students self-identified as right-handed, with normal or corrected to normal vision, and received a $10 gift card for each hour spent completing each of 15 sessions that lasted between 45min and 1h. Only a subset of the data, from three different two-button conditions are analyzed and presented here.

##### Procedure

The two-button data was collected in the context of a much larger study that collected data from 2, 4, 6, 8, and 10 button versions of the task (see Ma et al., 2016). The first five sessions administered the 2–10 button sessions in randomized order. All five sessions presented the trials with randomly varying ITIs, ranging from 820 to 1,420ms in 200ms increments. Next, participants completed five sessions that used a fixed 420ms ITI, followed by a tempo-based response deadline that was 100ms less than their mean for each of the standard 2–10 button tasks. The final five sessions imposed a response deadline that was 200ms less than their respective 2–10 button means in the standard, variable ITI version of the task.

The response tempo protocol was modeled on Kello and Plaut (2000), and implemented with a series of beeps, coordinated with the progressive subtraction of five left and right facing carets (>>>>> <<<<<) flanking the fixation boxes. The delay between each of five successive beeps was timed to be 100ms and then 200ms less than each individual participant’s standard two-button response time, as estimated by least-squares fit of their 2–10 button mean response time to Hick’s law. Participants were asked to synchronize their responses to occur in time with an unpresented final beep.

#### Results and Discussion

##### Tempo Hick Minus 200ms

**Figure 5:**
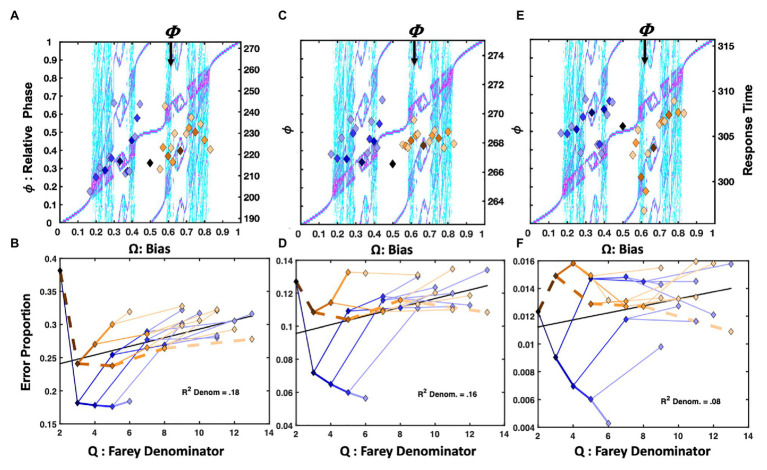
Plot (A) depicts normalized response times for the −200ms response deadline version of the two-choice Hick task. The X-axis is bias (**Ω**), and the left-hand *Y*-axis indicates relative phase (ϕ). The pattern is similar to that of the (Jones et al. 2013) repetition condition, in Figure 4A. Ratios <0.5, emphasizing the right hand are fastest, and drawn to staircase-coincident phase attractive regions; trial-history ratios >0.5 are compressed below the diagonal. Plot (B) depicts the corresponding error rates of the trial-history ratios in terms of their color-coded Farey branching relationships. While the −200ms deadline yielded relatively high error rates, the ratios closely tracked the Farey tree’s denominator-stability predictions. Plot (C), displays the normalized response times for the −100ms response deadline version of the task. In this case, they are comparable over the range of trial-history sequences. By contrast, in plot (D), the error rates again tracked the Farey branching and ratio denominators. Excepting the golden ratio path, the normalized response times in plot (E) are relatively similar. The marker dispersion is almost identical to the (Jones et al. 2013) alternation condition in Figure 4C. Apparently, the slower, variable ITI version of the task favored the intrinsic antiphase bimanual coordination attractor. The error rates indicate increasing stability for the golden ratio path (F). The stability advantage for repetition, evident in the previous versions of the task, is maintained, but more repetitions are required to achieve stability.

Only observations between 1 and 1,200ms were admitted for statistical analysis in all three versions of the Hick’s Law studies. Figure 5A depicts the normalized response times for the Hick task in which the response deadline was speeded by 200ms from each participant’s average two-button response time, as estimated in the standard Hick task. The trial-history scatter plot is strikingly similar to Figure 4A, depicting the response times from the Jones et al. (2013) repetition condition. The association between the staircase and the trial-history latencies was reliable, *F*(1, 29) = 10.28, *r^2^* = 0.26, *p* = 0.003. Just as for the Jones et al. (2013) data, the **RRRRR** pattern is fastest, and the **RLLLL** pattern is advantaged relative to its neighbors. However, the basis of the similarities for the **RLLLL** sequence is somewhat distinct. Self-resonance on the left index finger, albeit slower, resulted in speeding the **RLLLL** sequence in the Hick task. In addition to self-resonance, biased presentation rates also supported the performance in the Jones et al. (2013) task. Comparing the two task’s error rates on the **RLLLL** path makes this clear. In the speeded Hick task, errors increase as progressively more **L**s are added to the sequence. By contrast, the error rates decline as more **L**s are added to the same sequence in the Jones et al. (2013) task. This distinguishes the intrinsic dynamics from a learning effect since the **LLLLLL** pattern was overrepresented in the Jones et al. (2013) task (see Figure 4B).

Likewise, the golden ratio trial histories produced a slight advantage relative to immediate neighbors for the 200ms speeded Hick task and the Jones et al. (2013) repetition condition. Growing advantages on the golden ratio path (ending with: **RLRLR**) were similarly noted for the Jones et al. (2013) repetition condition, the Remington (1969) task, and the Kirby (1976) delayed ITI response times in Figures 3A,B, 4A respectively.

The error rates appear in Figure 5B. The Farey tree depiction revealed the repetition pattern was very stable, and enjoyed much lower error rates than the remaining trial histories. However, the golden ratio path, depicted with heavier dashed lines, was advantaged relative to its place in the Farey tree, and the remaining trial histories. The Spearman correlation with the Farey tree’s shape was otherwise, very strong *F*(1, 28) = 202.16, *r^2^* = 0.88, *p* < 0.001. The ratios on the golden ratio path accounted for several major deviations from the Farey tree shape. The speeded nature of the task yielded relatively large error rates overall. They were correlated reliably with the ratio denominators, *F*(1, 29) = 6.37, *r^2^* = 0.18, *p* < 0.02. As predicted by oscillatory theory, the path to the **RLLLL** sequence produced the most errors. This discrepancy illustrates that **R** repetition is qualitatively different than **L** repetition. The differential error rates establish **R** repetition as intrinsically more stable than the **L** repetition for right-handed participants.

##### Tempo Hick Minus 100ms

Figure 5C displays the results when the deadline was just 100ms less than each participant’s average two-button response time in the standard Hick task. In this condition, the response times are all relatively similar, and roughly aligned across the plot’s center line. The staircase and relative phases are not significantly correlated. By contrast, the error rates, in Figure 5D, conform well to the Farey tree, *F*(1, 28) = 122.94, *r^2^* = 0.81, *p* < 0.001. The correlation between the error rates and the ratio denominators is weaker, but remains statistically reliable *F*(1, 29) = 5.51, *r^2^* = 0.16, *p* < 0.03. Apparently, the repetition and alternation patterns are more stable than the remaining patterns, as their error rates tend to fall below their Farey tree expectations. From an oscillatory perspective, these particular advantages make sense. The repetition advantage arises because the dominant index finger increasingly behaves as an isolated oscillator with each repetition, and it enjoys the environmental support of button-press resonance, in addition to any potential physiological advantages that may arise in repeating similar motions. The alternation advantage emerges from its link to antiphase coordination, relative to the remaining trial-history ratios.

##### Variable ITI Hick

The top right plot, Figure 5E, depicts the results of the standard Hick task. Rather than a tempo-based timeline, it used variable ITIs and conventional instructions to respond as quickly and accurately as possible. In this case, the sequences on the golden ratio path to the alternation pattern display a clear response time advantage, relative to the remaining sequences. Again, the correlation between the staircase and trial-histories is absent.

An advantage for the repetition pattern persists in the Farey tree error plots. However, the alternation pattern again displays lower error rates than the expectation given by generic mode-locking. The correlation between the error rates and the Farey tree’s shape is reliable, *F*(1, 28) = 153.21, *r^2^* = 0.86, *p* < 0.001. Figure 5F illustrates their consistency with the Farey tree branching pattern. However, the error rates are very low, and the correlation between the error rates and the ratio denominators is not significant.

##### Summary

Looking across conditions reveals an apparent continuum from repetition dominance to alternation dominance in sequential effects. The two ends of the continuum closely resemble the impact of the bias effects observed by Jones et al. (2013). The Hick task also revealed an intermediate transition stage for which there was no clear response time advantage for any of the trial histories. Despite the absence of the effect on response time, the consistency of the error rate patterns across conditions, and with the Farey tree, implicate the −100ms deadline condition as an intermediate state in a transition from repetition to alternation dominance. The continuum illustrates the importance of the dynamic relations interfacing participant and task. Next, the impact of increasing cognitive load is explored.

## Persistence of Sequential Effects Under Cognitive Load

Sequential effects are commonly reported and studied in perceptual identification tasks. These tasks impose minimal cognitive loads and produce relatively brief response times. This section illustrates sequential dependencies consistent with the oscillatory model in slower, more cognitively demanding tasks. Each task uses a standard, balanced, two-alternative forced-choice paradigm.

The tasks were selected to form a loose rank-order of progressively increasing cognitive demand, as approximated by mean response time for correct, affirmative responses. An easy lexical decision task used unpronounceable, random letter strings as distractor items. In this task, correct responses averaged about 515ms. Substituting pronounceable nonwords as catch trials increased the mean correct word trials to 606ms. A third version of the task further increased difficulty by substituting pseudohomophones, again slowing correct word trials to 648ms. The fourth task was a classic letter rotation paradigm, modeled after Cooper and Shepard (1973). It presented individual letters that were rotated in 60° increments between 0° and 180°. It required longer affirmative judgments, averaging 1,268ms. On each lexical decision or letter rotation trial, participants pressed a “yes” (**R**) button for words or normally oriented letters, and “no” (**L**) for nonwords or mirror-reversed letters.

### Solo Lexical Decision and Letter Rotation

The aim of these four studies is to explore how coordination dynamics enfold with cognitive dynamics. From an oscillatory perspective, relative to the Jones et al. (2013) perceptual identification task, the easier lexical decision task slightly amplifies the frequency discrepancy between “yes” (**R**) and “no” (**L**) responses. When the discriminated items are distinct (e.g., larger *d'* or d-prime, a signal-detection sensitivity measure), and the task pace is relatively fast, the **R** and **L** frequency difference is small. This favors more stable entrainment with the driving signal. It also corresponds to a *weaker* coupling strength between **R** and **L** responding, allowing for faster yes and no responses and lower error rates. More difficult decisions (smaller *d'*) slow the task’s pace. If, as in lexical decision, recognized items must be discriminated from unrecognized items, it may also amplify the frequency discrepancy in **R** and **L** responses – a lexicality effect. Moreover, increasing ambiguity between target items and catch-trial items amplifies the **R** and **L** coupling strength (**K**). Ambiguity impedes the **R-L** symmetry break required for response selection.

In terms of predictions, increasing cognitive demand in a manner consistent with the existing entrainment patterns will exaggerate the magnitude of the raw response time differences in sequential dependencies, and increase compliance with the Devil’s staircase. By contrast, the increased error rates associated with more difficult decisions are predicted to decrease compliance with the driving signal, and thus, the Farey tree branching. On any given trial, the more coherent cognitive dynamics associated with recognition are expected to amplify performance speed and stability, while the less coherent dynamics associated with the absence of targeted stimuli are expected to erode performance speed and stability (Stone and Van Orden, 1994; Van Orden and Goldinger, 1994; Stone et al., 1997; Holden, 2002).

#### Method

##### Participants

For the lexical task, all participants self-identified as right-handed, native English speaking, with normal or corrected to normal vision, and participated as one of several routes to completing an outside of class research requirement. Data for 82 participants was collected for the easy lexical decision task. Data for 79 and 72 participants was collected for the hard and very-hard lexical decision tasks, respectively. The data collection activities were approved and overseen by the UC IRB. Data for 27 participants was collected in the letter rotation task. All participants self-identified as right-handed with normal or corrected to normal vision, and participated as one of several routes to completing an outside of class research requirement. The data collection activities were approved and monitored by the CSUN IRB. Distinct analyses of the rotation data, concerning letter rotation effects and distribution modeling, were previously reported in Ruzicka (2005) and van Rooij et al. (2013). Censorship criteria for each study were adjusted to admit similar proportions of participants, about 80%, in the statistical analyses. The goal was to impose an approximate *post hoc* control for participant-wise speed-accuracy tradeoffs across all three tasks.

##### Apparatus and Procedure

Each task was presented using a standard computer, screen, and keyboard. Targeted responses were identified with the participants’ right index fingers. On each *lexical decision* trial participants pressed a “yes” key if the presented was a legitimate English word, and a “no” key otherwise. The 1,100-trial task began with four buffer trials, and used letter strings ranging in length from four to six letters. The easier version used 548 random letter strings as distractors (e.g., *szbj*), the harder version used pronounceable letter strings as distractors (e.g., *talp*). The very-hard condition replaced 188 pronounceable nonword catch trials with pseudohomophones (e.g., *roap*). The 548 word-targets included a word-frequency manipulation. The *letter rotation* task was modeled on Cooper and Shepard’s (1973) paradigm. One of six characters (2, 5, 7, G, J, R) was presented on each trial. The characters were rotated at either 0°, 60°, 120°, or 180°. The orientations and character identities were balanced equally. Half the 1,680 trials presented normal characters, indicating a “yes” response, the remaining trials presented mirror-reversed characters and required a “no” response.

#### Results and Discussion

##### Easy Lexical Decision

**Figure 6:**
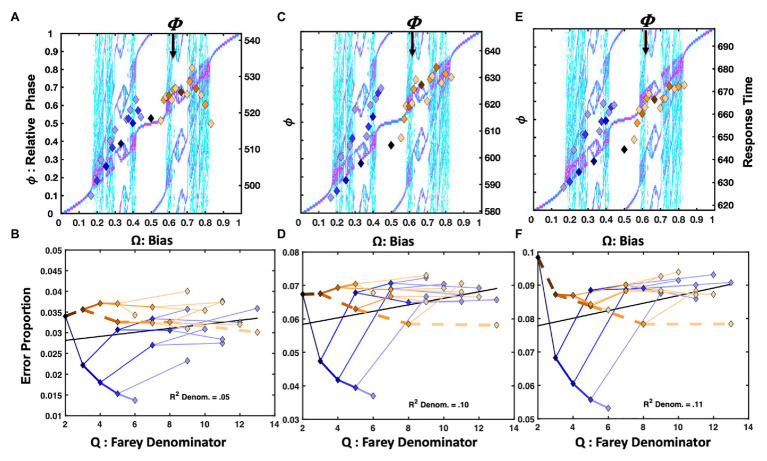
Plot (A) portrays the trial-histories for easy lexical decision as a function of the input bias (**Ω**) and their corresponding relative phase (ϕ). The association between the normalized response times and the staircase was strong, and several points that over- or undershoot the staircase are consistent with the bifurcation diagram’s phase attractive regions. Plot (B) indicates the error rates closely track the Farey branching. The harder lexical decision trial-histories appear on plot (C). Again, the correlation between the normalized latencies and the staircase was strong. Plot (D) depicts error rates for harder lexical decision as a function of the Farey tree branching. The association between the relative magnitude of the error rates and the Farey tree branching was robust. Plot (E) portrays the response times for the very hard version of the task; they closely tracked the staircase. The error rates in plot (F) illustrate a continued trend in which ratios with larger denominators garner more errors, while the stability of **R**-leading repetition and alternation is largely preserved.

The analyses only included data from 64 (78%) the 82 total participants who all produced overall error rates of 7.5% or less and maintained an overall mean response time of 800ms or less. Observations <250 or >1,200ms were censored from the analyses. Figure 6A depicts the easier lexical decision response times with the sequential dependency means plotted as points, over the bimanual model’s topology. The trial-history latencies reveal a close relationship with the Devil’s staircase, *F*(1, 29) = 52.68, *r^2^* = 0.65, *p* < 0.0001. The primary exception is an indication of self-resonance for the **RLLLL** sequence. Both the **RLLLL** pattern, its immediate precursor, **RLLL**, and its neighbor, **RLLLR**, fell below the staircase prediction. The apparent *absence* of a response time advantage for the golden ratio path, previously observed in the perceptual identification tasks, is also notable.

Figure 6B depicts the easy lexical decision error rates, in terms of their respective Farey tree ratios. Again, the error rates for each trial-history sequence are plotted with links between the values that track their respective branching connections on the Farey tree. The plot indicates a strong visual and statistical correspondence between the ratios and the shape predicted by the Farey tree branching. The correlation between the Farey tree prediction and the trial-history error rates observed in easy lexical decision is reliable, *F*(1, 28) = 386.20, *r^2^* = 0.93, *p* < 0.0001.

Notable outcomes are the much lower error rates associated with the repetition path. As in the perceptual identification tasks, the **RRRRR** pattern stands well apart from the remaining Farey tree branches, indicating comparatively robust performance stability. The **RLLLL** path displayed a modest speed advantage, but lacked the same stability in error rates that arose in the Jones et al. (2013) repetition bias manipulation. This difference in outcomes suggests the basis of the stability advantage achieved for the **RLLLL** pattern in the Jones et al. (2013) repetition bias manipulation was supported by longer-term learning-based changes.

The errors on the golden ratio path fell slightly below the location predicted by ratio branching indicated in a generic mode-locking Farey tree. This advantage likely results from the intrinsic stability of the golden ratio path, as it is phase attractive in bimanual coordination. Thus, compared to neighboring ratios, when participant’s responses were embedded in an alternation pattern they were better controlled and more dexterous.

However, the relative speed advantage for alternation, observed in simpler perceptual identification tasks is absent. Cognitive load is likely responsible for the absence of the speed advantage, given the apparent stability advantage indicated by the lower error rates. The **R** and **L** buttons are identified with word and non-word responses, respectively. Nonword responding is slower, as it is rooted in less coherent and less stable perceptual dynamics tracking learned relationships among spelling patterns, pronunciations, and semantics (Stone and Van Orden, 1994; Van Orden and Goldinger, 1994). However, for skilled readers, the statistical novelty of illegal nonword spelling patterns allows them to be quickly discriminated from words. The ~42ms lexicality effect effectively increased the frequency discrepancy between **R** and **L** index fingers used for responding. This yielded a slight increase in the duration of the **L** responses, and their mean relative to **R** responses. This, in turn, undermined the modest speed advantage for the golden ratio path observed in the faster perceptual identification tasks.

##### Harder Lexical Decision

The overall error rate and correct mean response time censorship criteria were adjusted to equate the proportion of censored participants with the easier version of the task. The analyses included data from 61 of the 79 participants (77%), who all produced error rates of 12% or less, and maintained an overall mean response time of 1,100ms or less. Observations <250 or >1,200ms were censored from the analyses.

Figure 6C depicts the bifurcation diagram for the harder lexical decision task. Again, the trial-history latencies closely track the Devil’s staircase, *F*(1, 29) = 96.68, *r^2^* = 0.77, *p* < 0.0001. Likewise, the scatter plot indicates the Farey ratios track the bimanual model’s relative phase topology as well. Deviations from the staircase pattern tend to be captured by nearby phase attractive regions.

The error rates, in Figure 6D, again closely track the Farey tree branching, *F*(1, 28) = 132.11, *r^2^* = 0.82, *p* < 0.0001. The notable exceptions are the trial histories on the golden ratio path. In this case, the intrinsic antiphase bimanual attractor offers performance stability second only to the repetition pattern. While the error rates generally respected the Farey tree structure, the remaining ratios were more homogenous. In terms of error-rate compression, the transition from easy to harder lexical decision mirrored the transition from the speeded to the standard Hick task. As the task’s pace slowed, the differences in the error rates associated with the various trial histories were compressed, and the error rate advantage for the golden ratio path grew.

The self-resonance advantage along the **RLLLL** path is absent for the harder version of the task. The pronounceable nonwords are more word-like, increasing the difficulty of discriminating words from nonwords. As noted, “no” responses rely on less coherent cognitive dynamics – an absence of recognition – and are thus generally slower than “yes” responses (Van Orden and Goldinger, 1994). Increasing the task difficulty increased the temporal discrepancy between **R** and **L** responses. The 110ms lexicality effect eroded the temporal advantage for alternation, but its lower error rates indicated its stability advantage remained intact. Apparently, differences in the relative coherence of recognized vs. unrecognized letter strings amplified the temporal discrepancies between **R** and **L** responses.

##### Very-Hard Lexical Decision

The analyses included data from all 72 participants since fewer individuals were recruited for the study. Observations <250 or >3,000ms were censored from the analyses. The trial history latencies, plotted on Figure 6E, are strongly correlated with the Devil’s staircase, *F*(1, 29) = 79.38, *r^2^* = 0.73, *p* < 0.0001. Likewise, the error rates, on Figure 6F, again correspond to the Farey tree, *F*(1, 28) = 75.20, *r^2^* = 0.73, *p* < 0.0001. Apparently, the **R**-leading repetition and alternation patterns are more stable in the face of increasing decision difficulty than the bulk of the remaining trial-histories. The error rates for the remaining trial histories tend to increase more as words and nonwords become harder to discriminate. Overall, the very-hard lexical decision continues the trends established in contrasts of the easy and hard versions of the task.

##### Letter Rotation

**Figure 7:**
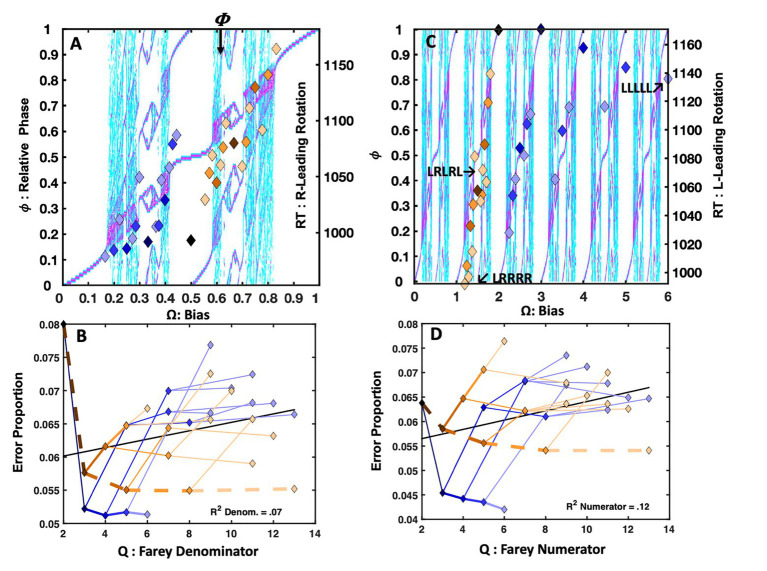
Plot (A) depicts the letter rotation trial-histories as a function bias (**Ω**) and relative phase (ϕ). The bulk of the trial-history ratios conformed to the staircase prediction. Plot (B) depicts the error rates in terms of the Farey tree branching. The error rates track the shape and details of the Farey tree, but not as well as for lexical decision. Instead, the **R**-leading repetition and golden ratio trial histories are more stable than the remaining sequences. Plot (C) depicts the normalized response times for the **L**-leading ratios, **L**, **LL**, **LR**, and so on. The location of three key trial-histories are highlighted on the figure. **L**-leading repetition **LLLLL** has among the largest relative phases, and **LRRRR** the shortest. The **L**-leading error rates generally conform to the **R**-leading Farey tree. Plot (D) indicates that **L**-leading repetition is the most stable ratio, and **L**-leading alternation is a close second.

The overall error rate censorship criterion was adjusted to eliminate participants returning overall error rates >17%. The analyses included data from 21 of 27 participants (78%), to approximate the speed-accuracy relationships returned by the lexical decision performances. The correlation between the normalized response times and the staircase was reliable, *F*(1, 29) = 160, *r^2^* = 0.85, *p* < 0.0001. The scatterplot appears in Figure 7A. The difference between normal and mirror-reversed trials was 188ms, about 71% larger than the hard lexical decision lexicality effect. Figure 7B illustrates the correspondence between the error rates and the Farey tree branching was weaker than for lexical decision, but nevertheless significant, *F*(1, 28) = 49.78, *r^2^* = 0.64, *p* < 0.0001. Notably, the error rate advantage for the golden ratio path in letter rotation is stronger than in lexical decision. The path’s stability, relative to ratios other than repetition, grew progressively with increasing lexical decision difficulty. This growth in relative stability continued to improve in letter rotation. Apparently, the relative stability of the golden ratio path increases as the task’s pace slows. Likewise, the bimanual coordination model’s antiphase attractor becomes increasingly stable as the driving frequency is lowered.

##### **L**-Leading Rotation Ratios

At this point, the **L**-leading ratios, complementing the **R**-leading letter rotation ratios, will be briefly discussed. The branching of an **L**-leading mode-locking Farey tree is a mirror image of the **R**-leading Farey ratios. Each **L**-leading fraction is the inverse of its **R**-leading compliment (e.g., 8/13 becomes 13/8). In fact, the Stern-Brocot tree encodes the entire **R**- and **L**-leading mode-locking structure. For example, the ratios for **L**, **LR**, and **LL** are 2/1, 3/2, and 3/1. As such, the **LRRRR** ratio of 6/5 is the smallest and **LLLLL**, or 6/1 becomes largest. This predicts the smallest relative phase for **LRRRR** and the largest for **LLLLL**; the opposite of the relative phase relations for **RRRRR** and **RLLLL**.

The **L**-leading ratio’s **Ω** range from 1.2 to 6, and the model’s dynamics repeat, or “wrap” for each integer increase in **Ω**. The **L**-leading letter rotation latencies are plotted in Figure 7C. For continuity, the color-coding is preserved from the previously presented **R**-leading plots, so tan ratios are smallest and blue largest. While the staircase’s unit-interval shape is preserved, the p/q axis increases by 1 for every unit increase in **Ω**. As Figure 7C makes clear, response times do not increase without bound. Apparently, the ratios are captured in the phase attractive region corresponding to the staircase’s attractor basins, across each successive unit-interval “wrap” in the bifurcation diagram. A correlation between a *modulo 1* version of the staircase ratios, where by convention 0 is shifted to 1, and the **L**-leading, unit-interval latencies indicates a strong association between the ratios and a “wrapped” version of the staircase, *F*(1, 29) = 117.67, *r^2^* = 0.80, *p* < 0.0001.

Figure 7D depicts the error rates as a function of the mode-locking ratios. Again, the **R**-leading colors are used. The outcomes are largely inverted relative to the predictions of the **L**-leading portion of the Stern-Brocot tree. The error rates are reliably correlated with the **R**-leading Farey tree, *F*(1, 28) = 100.29, *r^2^* = 0.79, *p* < 0.0001. Thus, the **L**-leading repetition pattern yields the smallest error rates, defining the plot’s lower boundary. By contrast, **LRRRR** is the most error-prone, and defines the upper boundary. As in the **R**-leading analyses, alternation displayed increased stability relative to all but the repetition pattern. At first, the fact that the **L**-leading error rates generally track the **R**-leading ratios is surprising. However, a close look at both the **R**- and **L**-leading error rates indicates intrinsic stability for both **R**- and **L**-leading alternation, and repetition for which extrinsic stability in the form of self-resonance is available. Otherwise, error rates dramatically increased for ratios that deviated from these core patterns, in both the **R**-leading and **L**-leading plots. Nevertheless, the response times correspond well with the predicted **L**-leading topology.

##### Summary

Overall, increasing cognitive load slowed performance and broadened the temporal range and potency of the sequential effects. Correct response times better approximated the Devil’s staircase as the tasks became more difficult. The contrast between the relatively more- and less-coherent cognitive dynamics entailed by recognition, and its absence, eroded the response time advantage for the golden ratio path. However, the error rates implicated the golden ratio path as fundamentally stable, relative to all other ratios, save for the repetition path. The stability of both the repetition and alternation ratios grew with cognitive demand, and the slowing pace of trials.

### Cognitive Demand

Apparently, increasingly stable intrinsic coordinative dynamics, in turn, stabilize cognitive performances. The essential evidence for this statement is the progressive decline in error rates associated with trial-histories on the intrinsically stable golden ratio path. Similarly, the repetition path, associated with self-resonance, illustrates that, other things being equal, consistent environmental entrainments also stabilize the faster within-trial dynamics of cognitive performance (Van Orden et al., 2012). Ultimately, these relationships are mutually reinforcing and reciprocal: coherent cognitive dynamics are also capable of amplifying and stabilizing action (e.g., Balota and Abrams, 1995; Moreno et al., 2011).

The results of Farey tree error rate analyses suggested the overarching impacts of adding cognitive load are both increases in the **R** to **L** coupling strength, that resemble the impact of adding noise to a dynamic system. Performance destabilizes in a manner respecting the relative coherence of the system’s own intrinsic dynamics. Repetition benefits more from environmental constraints, such as self-resonance, than alternation. Environmental support offers improved stability for repetition over alternation, except in the face of uncertainty in those constraints. Moreover, the error rates for trial ratios that branch from the repetition and golden ratio paths are very large, relative to branches remaining on the paths. This suggests cognitive acts that are commensurate with the repetition and the golden ratio paths are constrained by and stabilized by the immediate history and momentum of activity. Thus, sequential effects may relate to other historically significant developmental and cognitive phenomena, such as the A not B error (e.g., see Thelen and Smith, 1996; Erlhagen and Schöner, 2002) and stimulus-response compatibility (e.g., see Proctor and Reeve, 1989; Kornblum et al., 1990; Hommel et al., 2004).

#### Dis-Embodied Cognition

Both early and contemporary sequential effects research, noted in the introduction, must be credited with identifying the importance of repetition and alternation. However, the commitment computational theories make to symbolic, rational, and algorithmic Bayesian neural networks invite conceptual blind-spots that hamper scientific progress. For instance, if cognition is largely computational, why might two sequences that are equated for commonly exploited modeling variables, such as repetitions, alternations, and **R**-**L** letter counts produce latency differences? Differences between apparently comparable trial-histories such as **RLRR** and **RRLR** imply a need for more model parameters, such that ostensibly “predictive” models risk degenerating into case-by-case descriptions.

In fact, **RLRR** and **RRLR** do yield significantly different mean response times in letter rotation, *F*(1, 19) = 18.15, *r*^2^ = 0.48, *p* < 0.0001, **RLRR** *M* = 1,021ms, *SD* = 120ms, **RRLR** *M* = 982ms, *SD* = 122ms. The contrast was reliable for the hard lexical decision task as well, *F*(1, 77) = 7.06, *r*^2^ = 0.08, *p* < 0.001, **RLRR** *M* = 621ms, *SD* = 72ms, **RRLR** *M* = 617ms, *SD* = 71ms. From a computational perspective, the difference is puzzling but **RLRR** and **RRLR** correspond Farey ratios of 4/7 and 3/8, or 0.57 and 0.38, a contrast between a relatively slower and faster Farey ratio.

Computational theories such as Cho et al. (2002) and Jones et al. (2013) abstract sequential effects to pairwise counts of repetitions and alternations, and away from the specific trial-histories. As such, **LLLLL** and **RRRRR** response times are treated equivalently as repetitions, and averaged. From the perspective of coupled oscillators, these two patterns are distinct because **LLLLL** concerns a lower frequency oscillator, and **RRRRR** a higher frequency oscillator. Indeed, a contrast of the letter rotation repetition response times revealed a reliable response time difference between these two repetition sequences, *F*(1, 19) = 46.16, *r*^2^ = 0.70, *p* < 0.0001, **LLLLL** *M* = 1,136ms, *SD* = 158ms, **RRRRR** *M* = 934ms, *SD* = 145ms. However, their respective Farey ratios of 6/1 and 1/6 explain the difference.

Likewise, conventional accounts treat **R**- and **L**-leading alternation as identical patterns. However, the bimanual coordination literature established asymmetric limb coupling-strength in right-handed individuals. Their right hands entrain their left hands more effectively than their left hands entrain their right hands (Peper et al., 1995; de Poel et al., 2007). This predicts that **L**-leading alternation should be slower than **R**-leading alternation. Indeed, **LRLRL** is slower than **RLRLR** for letter rotation, *F*(1, 19) = 17.38, *r*^2^ = 0.46, *p* < 0.0001, **LRLRL** *M* = 1,072ms, *SD* = 139ms, **RLRLR** *M* = 1,027ms, *SD* = 115ms. Again, the respective Farey ratios of 13/8 and 8/13 explain the difference.

Both contrasts replicated on an identical analysis of the hard lexical-decision data; *F*(1, 77) = 97.07, *r*^2^ = 0.56, *p* < 0.0001, **LLLLL** *M* = 645ms, *SD* = 96ms, **RRRRR** *M* = 577ms, *SD* = 68ms. Again, **LRLRL** is slower than **RLRLR**, *F*(1, 77) = 18.72, *r*^2^ = 0.20, *p* < 0.0001, **LRLRL** *M* = 641ms, *SD* = 68ms, **RLRLR** *M* = 630ms, *SD* = 76ms. The results of these illustrative “mirrored” **R**- and **L**-leading letter rotation and lexical decision contrasts contradict predictions of computational theories that treat **R**- and **L**-leading repetition and alternation trial-histories as interchangeable (e.g., Cho et al., 2002; Jones et al., 2013).

Equivalent distinctions hold at the level of two-trial repetition (**RR** and **LL**) and alternation (**RL** and **LR**) counts, commonly described in the neural network modeling literature on sequential effects. Several **R**-leading ratios have equal repetition and alternation counts, but their Farey ratios explicitly predict response time differences. For example, **RLLLR** and **RRRLR** are comprised of two repetitions and two alternations each. Thus, conventional models predict comparable response times for these two trial-histories. However, the bimanual model predicts longer mean response times for **RLLLR** than for **RRRLR** since their Farey ratios are distinct: 7/9 (0.78) and 3/11 (0.27), respectively. A mean response time contrast of the rotation data verified the difference predicted by oscillatory dynamics, *F*(1, 19) = 39.93, *r*^2^ = 0.67, *p* < 0.0001, **RLLLR** *M* = 1,065ms, *SD* = 148ms, **RRRLR** *M* = 962ms, *SD* = 123ms. The same contrast on the hard lexical decision data was also reliable, *F*(1, 77) = 87.07, *r*^2^ = 0.53, *p* < 0.0001, **RLLLR** *M* = 640ms, *SD* = 78ms, **RRRLR** *M* = 607ms, *SD* = 67ms.

In another example, the **RLLRR** and **RRLRR** trial histories have equal repetition and alternation counts, but distinct Farey ratios, 7/10 (0.70) and 4/11 (0.36). Again, they have reliably different mean response times in letter rotation *F*(1, 19) = 16.15, *r*^2^ = 0.45, *p* < 0.0001, **RLLRR** *M* = 1,031ms, *SD* = 121ms, **RRLRR** *M* = 982ms, *SD* = 118ms, and hard lexical decision, *F*(1, 77) = 41.08, *r*^2^ = 0.35, *p* < 0.0001, **RLLRR** *M* = 731ms, *SD* = 74ms, **RRLRR** *M* = 611ms, *SD* = 72ms. Overall, discriminating the trial histories in terms of the individual Farey ratios given by oscillatory theory generates and supports many more detailed predictions than the conventional practice of counting the repetitions, alternations, or letter types for a given trial-history token. Furthermore, the fact that most contemporary theories average mirror-image trial histories (e.g., **RRRRR** and **LLLLL**, **RLRLR** and **LRLRL**, and so on) implies some previously modeled data inaccurately portray the empirical phenomena that are characteristic of sequential effects. Even if the aforementioned catalog of differences seems psychologically and computationally vexing, it is easily explained by basic principles of oscillatory entrainment. The Farey ratios correspond to more and less stable entrainment patterns with distinct relative phase relations, or characteristic oscillatory frequencies. Their relative stabilities arise from self-sustaining resonant feedback that emerges across trials.

Contemporary sequential effects models such as Cho et al. (2002) and Jones et al. (2013) appear to succeed because they are incrementally adaptive. Notably, adaptive sequential dependency models typically capture nearly 100% of the response time variance. This points to a fundamental limitation of adaptive models: they capitalize on extant autocorrelations in the data set, and reproduce, rather than explain them. They effectively demonstrate that one may apply probabilistic supervised learning techniques to approximate sequential effects. However, they must adopt various hypothetical (unobserved) cognitive faculties that explicitly build-out a probabilistic and mathematically complex history-tracking system. Presumably, these systems can be tuned to track and reproduce many deterministic sequential dependency patterns, even those that are never observed.

#### Embodied Cognition

By contrast, an oscillatory account leverages long-established facts relating to the coordination of bodily dynamics and related neurophysiological states in its account of performance. For example, the success of the oscillatory predictions illustrates how the immediate physical history of the body influences cognitive activity, and serves as an environmentally extended, embodied, and dynamic form of short-term memory (Hutchins, 1995; Chemero, 2011). That is, it acknowledges that the neurophysiological, bodily, and physical consequences of previous acts support new acts (Michaels and Carello, 1981; Turvey and Carello, 1986; Turvey, 2007, 2018; Turvey and Fonseca, 2014). As such, these states needn’t be explicitly constructed, stored, tracked, or represented in the mind, because they are physically and dynamically manifested, and thus provide ongoing constraints for every new physical and intellectual act (Gibbs, 2005; Chemero, 2011; Anderson et al., 2012; Van Orden et al., 2012; Wijnants et al., 2012).

Nevertheless, it is important to avoid framing the relative success of either the oscillatory and learning/memory accounts as a winner-take-all modeling contest. Seeking to pit intention, learning, and/or memory against oscillatory dynamics in mutually exclusive hypothesis tests is misguided; intention, learning, memory, and oscillatory entrainment do not preclude each other. Instead, their roles may be disentangled in terms of their characteristic timescales of action. For instance, oscillatory entrainment likely governs the fastest performance timescales, from milliseconds to several seconds – it serves as a foundational constraint for participants as they tune their performances across longer timescales, and more trials. Thus, influences relating to intention, memory, and learned expectations may become increasingly important over longer timescales of action. Moreover, the long-term performance impacts of learning, memory, and intention are already well established (e.g., Kirby, 1976; Luce, 1986; Cho et al., 2002; Jones et al., 2013). The oscillatory theory simply offers an embodied broadening for the scope of more immediate performance constraints (Van Orden et al., 2012; Wijnants et al., 2012).

By this account, successful forced-choice performance requires an ongoing, parallel resolution among many competing demands presented by a task’s “atoms”: the myriad variables, processes, and constraints related to perception, discrimination, and response formation, alongside supporting background variables relating to relevant knowledge and experience, and immediate circumstances, such as gaze, posture, goals, and so on (see Mitra et al., 1998). For example, the inclusive complexity of a two-choice decision state-space is evidenced by the prominent, and contextually varied role that coordination dynamics play in the range of decision performances described here. Furthermore, each task is drawn from a psychological subdiscipline, such as word recognition, that itself involves many widely studied factors impacting performance (e.g., lexicality, word frequency, consistency, etc.). This illustrates the difficulty in identifying concrete material loci for many cognitive faculties involving learning, memory, and recognition. These capacities are recruited on-line, in ongoing pattern formation and self-organization processes (Van Orden et al., 2003a; Turvey, 2007, 2018). The roles and the functional states of their physiological, behavioral, and bodily correlates continually fluctuate, adapt, and re-organize across a range of timescales (Liu et al., 2019).

Thus, skilled performance emerges as participants gradually tame many competing demands by discovering and tuning their acts to the most functionally salient variables while resolving, enfolding, coordinating, and leveraging any support offered by the remaining task atoms. In this way, a previously high-dimensional state-space is re-organized into a compressed, lower-dimensional collective variable – a coordinative synergy – a softly assembled, self-organized coordinative structure that is revisited and adapted as needed (e.g., see Turvey and Carello, 1996; Mitra et al., 1998; Van Orden et al., 1999; Turvey, 2007; Holden and Rajaraman, 2012).

The next study underscores how dynamic relations, established by the interface between participants and task requirements, more strongly constrain observed performances than the base probabilities of various stimulus-response relations. A multi-stimulus extension of the standard letter rotation paradigm manipulates the relative frequency (mean latency) of the **R** and **L** or affirmative and negative responses. It is expected to dictate the sequential dependency patterns observed in response time. By allowing for progressively faster negative responses, the golden ratio path is predicted to recover its response time advantage under cognitive load, and eventually invert the sequential dependencies in response time, so the **L**-leading trial histories correspond to the **R**-Leading staircase, and the **R**-leading ratios track the **L**-leading modulus 1 staircase.

## Controlling Dynamics with Characteristic Response Frequencies

The previous section revealed how increasing cognitive demand diminished the response time advantage for the golden ratio path. Neither the lexical decision nor the letter rotation studies revealed a golden ratio response time advantage. Nevertheless, a golden ratio advantage was progressively amplified in error rates. The working explanatory hypothesis was that the increasing effect-size of the cognitive manipulation increased the characteristic frequency difference between the **R** and **L** response durations. This eroded the temporal advantage supplied by the antiphase attractor intrinsic to bimanual coordination. A stability advantage, however, was still apparent in the reduced error rates associated with the golden ratio path. As the typical **L**-trial slowed, the stability of the golden ratio path, as estimated by within-task relative error rates progressively approached the repetition error rate levels.

Oscillatory theory predicts that dynamical relations, established by task demands, will dictate the emergence of sequential effects. Essentially, the multi-stimulus rotation task progressively *increases* the characteristic frequency of **L** responses relative to **R** responses by allowing progressively faster “no” responses. Since the golden ratio path is on the right half of the Devil’s staircase, at some point, a response time advantage for the golden ratio may reemerge. Moreover, if the response time advantage for the **L** responses grows large enough, then the sequential dependency pattern has the potential to reverse direction. At that point, performance on **R**-leading ratios should resemble the **L**-leading relative phases, and the **L**-leading ratios should approximate the **R**-leading staircase. As it happens, both predictions are borne out in the empirical patterns.

### Stacked 1-2-4-8 Letter Rotation

On each trial participants discriminated normally oriented letters from mirror-reversed counterparts in a vertical column. They were instructed to press a “yes” key if all the presented letters were normally oriented, and a “no” key otherwise. The crucial aspect of the design is that “no” responses will tend to become faster than “yes” responses as the number of characters grows. A “no” response can be registered as soon as a reversed character is encountered. Since they are equally likely in all positions, on average, “no” responses take about half as long as yes responses, as all the positive trials require an exhaustive evaluation. All the letters were subject to rotations that varied randomly in 60-degree increments from 60° to 300°.

#### Method

##### Participants

A total of 17 undergraduate students self-identified as right-handed, with normal or corrected to normal vision, and participated as one of several routes to completing an outside of class research requirement. Ten participants completed the 1-2-4 item condition, and seven completed the 2-4-8 item condition. The data collection activities were approved and monitored by the CSUN IRB.

##### Design and Procedure

On each trial, participants discriminated between normally oriented and mirror-reversed versions of a set of alphanumeric characters (2, 4, 5, 7, F, G, J, L, P, R). The letters were visually presented in vertical stacks of 1, 2, 4, or 8 characters. The letters were rotated unsystematically in 60-degree increments ranging from 0° to 300°. Participants pressed the “yes” key with their right finger if all the characters were normally oriented, and the “no” key with their left finger otherwise. Half the trials were negative catch-trials and contained a single mirror-reversed character in one of the available character slots. The mirror-reversed characters were evenly distributed across all available slots. One group of participants saw a balanced set of 792 trials with equal proportions of letter stacks comprised of one, two, or four characters – the 1-2-4, condition. A second group of participants completed 768 trials of an identical task that was comprised of a balanced set of 2, 4, and 8 characters – the 2-4-8 condition.

#### Results and Discussion

Imposing a 15% or less total error rate disqualified two of the 10 participants from analysis of the 1-2-4 condition. Imposing same error criterion, and an average response time of 3,750 or less on the 2-4-8 condition yielded four data sets for analysis.

##### One-Two-Four Rotation

**Figure 8:**
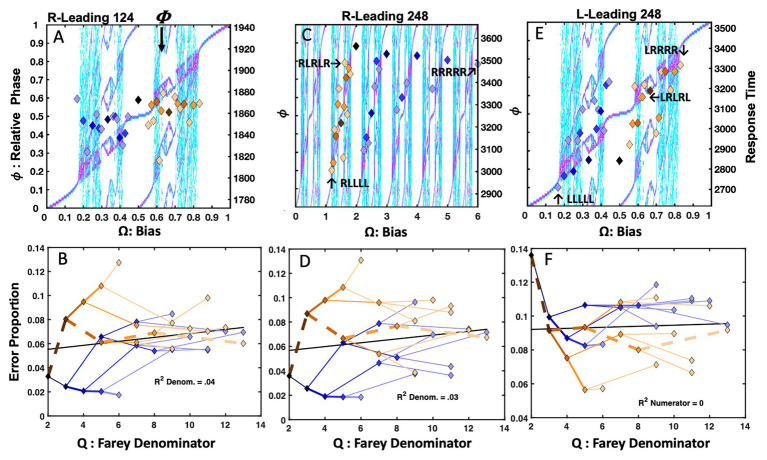
Plot (A) depicts the 1-2-4 letter rotation trial histories as a function of bias and relative phase. The repetition and golden ratio trial histories were faster than the remaining sequences. Plot (B) depicts the error Farey tree for the 1-2-4 condition. It retained consistent branching, despite the manipulation’s impact on the relative speed of **R** and **L** responses. Plot (C) depicts the **R**-leading 2-4-8 letter rotation trial histories, in terms of the **L**-leading ratios and bifurcation diagram. Increasing the duration of “yes” responses slowed the **R**-leading ratios, and they expressed the relative phases typical of the **L**-leading ratios. Plot (D) indicates the error branching remained consistent with the stability of the **R**-leading ratios. Plot (E) depicts the **L**-leading ratios for the 2-4-8 task. The speeded “no” responses conformed to the staircase, as predicted by their new role as the relatively faster oscillator. In Plot (F) the color-coded Farey branching for the **L**-leading ratios is inverted in a manner consistent with the **L**-leading portion of the Stern-Brocot tree. The **LRRRR** pattern, and the related brown and tan branches are the least error prone. The **LLLLL** pattern has lower errors, relative to related branches, indicating stability. Likewise, the bulk of the blue branches now fall above the regression line.

Figure 8A depicts the response time results for the 1-2-4 Letter rotation condition. In this case, response times for the bulk of the trial-history sequences are relatively similar. The correlation between the staircase and the trial-history response times was not significant. However, building faster “no” responses into the task allowed the golden ratio to recover its response time advantage, relative to performance for the neighboring ratios, lexical decision, and the single letter rotation task. Figure 8B illustrates the error rates in terms of their links among the branches of the Farey tree. Again, the error rates conformed to relative stability predictions intrinsic to the shape of the Farey tree, *F*(1, 28) = 413.90, *r^2^* = 0.94, *p* < 0.0001. Introducing the opportunity for faster negative responses tended to re-equalize the response times. Moreover, the underlying stability patterns predicted by oscillatory theory remained intact.

##### Two-Four-Eight Rotation

The 2-4-8 rotation response time results appear in Figure 8C through Figure 8F. Since affirmative **R**-responses required an exhaustive evaluation, the 2-4-8 version of the task further increased **R**-leading response times relative to negative **L**-leading responses. The manipulation was sufficient to entrain the **R**-leading performances to the **L**-leading *modulo 1* staircase, as depicted in Figure 8C, *F*(1, 29) = 71.66, *r*^2^ = 0.71, *p* < 0.0001. Figure 8D illustrates the error rates in terms of their Farey tree branching. Despite the response-time alignment with **L**-leading staircase, the error rates conformed to the stability predictions of the **R**-leading Farey tree, *F*(1, 28) = 468.45, *r^2^* = 0.94, *p* < 0.0001. Thus, affirmative responses maintained their **R**-leading stability properties.

##### **L**-Leading Ratios

Latencies for **L**-leading ratios aligned with the staircase. Figure 8E indicates the relationship between the **L**-leading performances and **R**-leading staircase was robust, *F*(1, 29) = 95.38, *r*^2^ = 0.77, *p* < 0.0001. Exchanging the typical durations of the “yes” and “no” responses resulted in ratios >1/2, that are typically slowest, such as **RLLLL** and neighboring ratios, to be generally faster than ratios <1/2. The **L**-leading error rates corresponded with the **L**-leading Farey ratios, *F*(1, 28) = 227.20, *r^2^* = 0.89, *p* < 0.0001. This pattern is visually evident in Figure 8F. The color coding from the **R**-leading branching was preserved, and is inverted, relative to the other Farey tree branching depicted in Figure 8D. Thus, while shortening the typical duration of “no” responses allowed a speed advantage to emerge, the stability inherent in pairing “yes” responses with the dominant hand, and “no” responses with non-dominant hand persisted.

##### Summary

Overall, the outcomes illustrate how the response times associated with the various trial-histories are quite sensitive to methodological circumstances and task requirements. Extending the duration of **R** responses relative to **L** responses allowed the reemergence of the golden ratio response time advantage in the 1-2-4 condition. The 2-4-8 condition further exaggerated the difference between the **R** and **L** responses, so that “yes” responses conformed to the **L**-leading staircase, and “no” responses conformed to the **R**-Leading staircase.

On the other hand, the error rates remained largely consistent with the mode-locking Stern-Brocot tree. This suggests one may reframe the classic speed-accuracy trade-off in dynamical terms. The relative speed of responding must be controlled on the very fast within-trial timescale. By contrast, accuracy is *course-grained* relative to speed, since individual trials represent the fastest accuracy timescale, or grain size (see Liu et al., 2019). Put differently, error variability is a consequence of incorrect acts, response time differences arise in the variable timing of correct acts. If the response times are speeded at the expense of the slower timescale constraints entailed by the appropriate response act, the error rates would simply explode. This again illustrates how stability on a slower timescale supports the dexterity and coordination needed for accelerated responding (Turvey, 2007; Van Orden et al., 2012; Wijnants et al., 2012). The delicately balanced relationship between speed and stability can be understood in terms of the interplay across the typical grain-size and timescales of the performances. A traditional speed-accuracy trade-off function approximates these boundary conditions across manipulations and participants.

If nothing else, the 1-2-4-8 rotation results underscore the need for theories and explanations of cognitive phenomena to be framed in terms of dynamic relations established by the participants’ goals, aptitudes, and task demands, rather than in terms of the properties of those task-atoms, *in and of themselves* (Mitra et al., 1998; Van Orden et al., 2003a; Turvey, 2007). Next, dyadic lexical decision, in which the word-nonword decisions are distributed across two individuals, is presented. The study was designed to test for sequential dependencies arising from spontaneous entrainment.

## Distributed Cognition

Computational sequential effects models are framed in terms of the probabilistic history of processed stimuli and responses executed by a single individual. The quantities required for tracking and learning trial-histories are said to be captured by brain-based neural networks, memory traces, and similar event-related representations in motor cortex (e.g., see Jentzsch and Sommer, 2002; Jones et al., 2013). In the absence of additional *post hoc* assumptions, standard computational sequential effects models predict an absence of sequential dependencies in dyadic performances. Each participant responds with a single finger, to a single type of stimulus. As such, each individual’s base response rate is 100% in favor of a single stimulus and response type. Effectively, each individual performs only a part of the task, and the extant models do not encode any obvious mechanism for the required probabilities to be computed and exchanged between two individual’s separate motor cortices.

By contrast, oscillatory theory predicts the emergence of spontaneous oscillatory entrainment that is distributed across separate individuals and consistent with the details of oscillatory dynamics, the HKB model, and interpersonal coordination findings (e.g., Marsh et al., 2009; Amon et al., 2018). The propensity for entrainment in informationally coupled oscillatory systems is well established (e.g., Huygens, 1673/1986). As such, the oscillatory framework predicts that dyadic performances should largely mirror individual performances.

### Dyadic Lexical Decision

A final lexical decision study delegated word and nonword responding between two separate individuals. They were seated together in front of a single PC and keyboard. One participant was assigned the affirmative “word” **R** responses, the other was assigned negative “nonword” **L** responses. Presumably, they would couple informationally, by the shared goals and context entailed in the task’s requirements. Otherwise, the task was equivalent to the single-person lexical decision task procedures described previously in the cognitive load section.

#### Method

##### Participants

All participants self-identified as right-handed, native English speaking, with normal or corrected to normal vision, and participated as one of several routes to completing and outside of class research requirement. Ninety-six undergraduate students participated in dyads to complete one of three versions of the lexical decision task. The specific dyadic parings were determined by the appointment time-slots in which participants chose to serve in the study.

##### Design and Procedure

The primary manipulation was the relative difficulty of the lexical decisions. In this study, three versions of the lexical decision were presented: easy, hard, and very-hard, as in the earlier lexical decision studies. The very-hard condition replaced 24% of the 550 pronounceable nonwords with pseudohomophones (e.g., *brane*). The target word and nonword lists were different than those used for the two studies presented earlier. On any given trial, the participant on the right was instructed to press the right-shift key if the presented letter string was a word, and the left-side participant was instructed to press the left-shift key if the presented letter string was not a legitimate English word. Unfortunately, fifty word-targets were repeated in the very-hard condition, due to a programing error; all but seven were low frequency items. Any repetition influence was minor, since the error respected the crux word-nonword distinction. The easy and hard conditions used a 250-1,200ms censorship boundary. The very-hard condition used 250 to 2,500ms censorship boundary.

#### Results and Discussion

##### Dyadic Easy Lexical Decision

Thirty-eight participants were paired into 19 dyads that completed the easy condition. One dyad reversed their word-nonword roles, relative to their assigned keys. We subsequently inverted and included their responses in the analysis. The trial-histories were correlated with the staircase, *F*(1, 29) = 50.30, *r^2^* = 0.63, *p* < 0.0001. This outcome is nearly identical to that observed for easier lexical decision in individuals. As for the solo version of the task, both the **RLLLL** pattern, its immediate precursor, **RLLL**, and its neighbor, **RLLLR**, fell below the staircase prediction. Again, the response time advantage for the golden ratio path is absent, as was previously observed for the solo version of the task.

**Figure 9:**
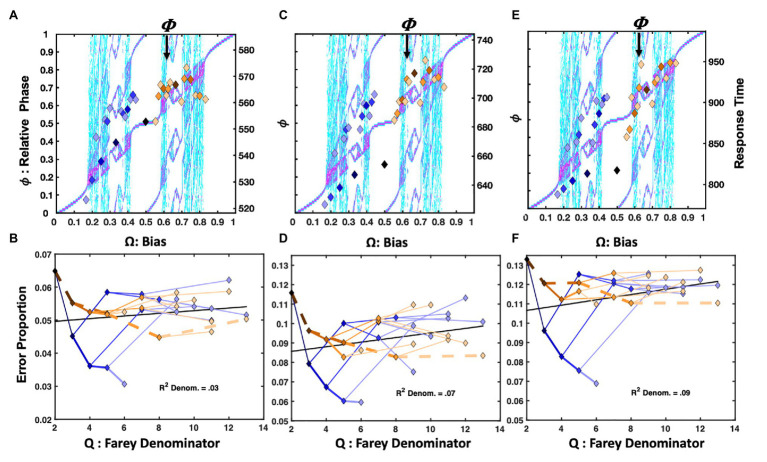
Plot (A) depicts the normalized response times for the dyadic easy lexical decision condition. The trial histories express some compression for the sequences on the **RLLLL** path. Also, the ratios between 0.25 and 0.5 tend to occupy phase attractive regions just above the diagonal. These outcomes closely mirror the solo version of this task depicted in Figure 6A. The dyad’s error rates appear on plot (B) as a function of their Farey tree branching. The repetition pattern associated is with self-resonance and favored relative to the other ratios. Likewise, the alternation pattern associated with the antiphase coordination was favored relative to nearby ratios. The response times for the hard lexical decision appear in Plot (C). The trial histories are again strongly associated with the Devil’s staircase. The corresponding error rates are depicted in plot (D), note the *Y*-axis expansion, relative to Plot (B). Again, the self-reinforcing repetition and the alternating golden ratio path are favored relative to the bulk of the remaining ratios. Likewise, the same basic outcomes were evident for the very-hard response times and errors appearing in plots (E,F).

Visually, the pattern depicted in Figure 9A is also similar to that of the single-person version of the task, plotted in Figure 6A. As noted, the larger ratios associated with the **RLLLL** path are compressed, but less so than for the solo version of the task. Overall the raw dyadic response times are slightly delayed, relative the solo task. Figure 9B depicts the error rates in terms of the Farey tree branching. Both the repetition and golden ratio paths are advantaged. Otherwise, the error rates again closely track the Farey tree branching, *F*(1, 28) = 77.03, *r^2^* = 0.73, *p* < 0.0001. Overall, the dyadic easy lexical decision performances closely mirrored those of the solo version of the task.

##### Dyadic Hard Lexical Decision

Twenty-four participants were paired into 12 dyads that completed the hard condition. As in the solo version of the task, the sequential dependencies closely corresponded to the Devil’s staircase prediction, *F*(1, 29) = 70.87, *r^2^* = 0.71, *p* < 0.0001. As for the solo task, dyadic staircase compliance was enhanced, relative to the easy condition. Again, the scatter plot of the sequential dependencies depicted in Figure 9C echoes that of the single-person version of the task, plotted in Figure 6C. The response time advantage for the golden ratio path is absent, and weak compression in the **RLLLL** path persists.

Figure 9D depicts the dyadic error rates in terms of their location on the Farey tree. Again, the self-resonance associated with repetition, and the golden ratio path are more accurate, or stable, than the remaining trial histories. The remaining error rates closely track the Farey tree branching, *F*(1, 28) = 60.34, *r^2^* = 0.68, *p* < 0.0001. Again, both the dyadic response time and error rate measurements closely resembled those of the solo versions of the task.

##### Dyadic Very-Hard Lexical Decision

Thirty-four participants were paired into 17 dyads that completed the very-hard condition. As with the other lexical decision tasks, the trial-history ratios were strongly associated with the Devil’s staircase, *F*(1, 29) = 83.71, *r^2^* = 0.74, *p* < 0.0001. Figure 9E depicts the dyadic very-hard lexical decision error rates in terms of their location on the Farey tree. As in all the previous tasks, the self-resonance associated with repeated affirmative responses yielded the lowest error rates. Likewise, the golden ratio path was more stable than its neighboring trial histories. Figure 9F indicates the remaining error rates closely track the Farey tree branching, *F*(1, 28) = 92.91, *r^2^* = 0.77, *p* < 0.0001. The results of the dyadic version of the very-hard task are in-line with the continuum of performance established by the solo versions of lexical decision. Overall, the distributed decision tasks gave rise to coordinative activity that closely resembled the solo tasks, and the patterns of spontaneous entrainment typical of social coordination, more generally (e.g., Marsh et al., 2009).

Finally, the staircase defines the model’s attractors. However, across the cognitive studies in the sections Persistence of Sequential Effects Under Cognitive Load and Distributed Cognition, some trial-histories occupied alternative phase attractive regions on the bifurcation diagram. Thus, one might reasonably ask if the attractors are predictive, beyond the bifurcation diagram’s circular means (Circular means respect the adjacency of 0 and 1 on the map). A multiple regression on the single-letter rotation and all six lexical studies, with both predictors, captured 70% of the trial-history variance *F*(2, 214) = 249.92, *r^2^* = 0.70, *p* < 0.0001. Subsequent partial regression analyses indicated the staircase captured 30% of the variance not associated with the bifurcation diagram means. By contrast, the map’s circular means captured only 5% of the variance not associated with the staircase *F*(2, 215) = 107.58, $pr_{DS}^{2}$ = 0.30, *p* < 0.0001 and *F*(2, 215) = 17.93, $pr_{CM}^{2}$ = 0.05, *p* < 0.0001. This is consistent with the article’s core narrative that the Farey ratio’s attractor status is the principal basis for the observed patterns, but the map’s various phase attractive regions can influence observations.

##### Summary

Apparently, cognitive load slows manual responding by enhancing **R** and **L** limb coupling, and lowering the task’s overall oscillatory frequency. This finding reveals a fundamental difficulty in segregating movement dynamics from cognitive dynamics. The fact that distributed performances preserve the coordinative dynamics of solo performances underscores this difficulty. On the other hand, the explanatory power of coordinative principles to generalize over a variety of laboratory-based decision tasks, and even to distributed cognitive systems promises a more unified scientific characterization of decision performance.

## General Discussion

Reframing response time performance in oscillatory terms allowed for a straightforward explanation of sequential effects as arising from oscillatory entrainment. The oscillatory principles entailed in the HKB bimanual coordination model explained several historically studied sequential dependency patterns. It organized all 31 of the classically studied **R**-leading one- to five-trial histories in terms of the Devil’s staircase. Each trial history was identified with a bias (**Ω)**, and a predicted mode-locking Farey ratio (p/q), corresponding to a unit-interval normalized mean response time.

The bimanual model’s oscillatory dynamics predicted a host of the coordinative relationships observed in generic response-time and error-rate sequential effects. The response time trial-histories from several historical and contemporary perceptual identification tasks closely tracked the Devil’s staircase predictions. The relative error susceptibility, or conversely the relative stability of the various trial histories was predicted by Farey tree branching. The enhanced stability of antiphase bimanual coordination afforded the most pervasive golden-ratio exception to generic Farey tree branching patterns. Ancillary analyses illustrated how the bimanual model’s predictions generalized to relative phase relations observed for the 31 **L**-leading trial-histories.

The tempos and typical time-courses of the tasks influenced the expression of the sequential dependency patterns. Tasks with a faster pace (e.g., small ITI) and minimal cognitive load favored conditions for self-resonance, and an advantage for responding that entailed repetition. Slower pacing and low cognitive loads allowed the golden ratio path to gain a foothold and display an advantage relative to neighboring trial histories. Moreover, the sequential effects persisted under cognitive load, and were influenced by altering the typical latencies of “yes” and “no” responses. They also generalized to dyadic decisions. In oscillatory terms, these manipulations influenced coupling strength and the characteristic frequencies of affirmative (**R**) and negative (**L**) responses.

The successful generalization of the bimanual model’s dynamics to explain sequential effects demonstrates a much tighter coupling between cognition and action than is predicted by conventional computational and modular theories of cognition and decision-making (Holden et al., 2011; Holden and Rajaraman, 2012). For example, the relative error rates were exquisitely conditioned by the array of trial-histories, and strongly correlated with mode-locking predictions. Moreover, the tendency for **R**-leading repetition to separate from the remaining ratios reveals a resonance advantage when strongly coherent cognitive attractors are supported, reinforced, and amplified by favorable and consistent environmental circumstances.

### Cognitive Dynamics and Control

In addition to supplying a plausible explanation for the basis of sequential effects in response time, the study outcomes also contribute to a broader narrative on the role of oscillatory dynamics in cognitive performance. Previous studies established that participants spontaneously entrain to oscillatory patterns in the timing and structure of elementary detection and production tasks such as simple response time, temporal estimation, and manual tracking (e.g., Peper et al., 1995; Holden et al., 2011; Amon et al., 2018; Jagacinski and Flach, 2018). The discovery that oscillatory dynamics play foundational roles in sequential effects and are leveraged by participants to accommodate task requirements implies an oscillatory etiology for 1/ƒ scaling in response time measurements: stochastic entrainment across the many time scales of change in physiological, cognitive, bodily, and environmental circumstances (Kello et al., 2007). This self-similar 1/ƒ, or fractal scaling is symptomatic of multiscale coordinative activity that emerges as a consequence of ongoing cooperative and competitive influences in biological, physiological, cognitive, and bodily processes that are all required to balance the ongoing demands of changing environmental circumstances and metabolic requirements (Buzsáki, 2006; Turvey, 2007; Turvey and Fonseca, 2014; Liu et al., 2019).

The Farey ratio’s sequential effects are available in response time analyses because they unfold repeatedly across trials, on a timescale slow enough to be captured by the statistical tools and methods of behavioral studies. As such, the oscillatory analyses reveal *momentum* in perception, action, and cognition. Particular trial-histories supplied predictable constraints to upcoming performances. Thus, performance in the present moment is always nested within constraints supplied by historical circumstances (Michaels and Carello, 1981; Van Orden et al., 2003b, 2012; Holden et al., 2009; Wijnants et al., 2012).

Response time measurements are sandwiched between the fast timescales of change in the nervous system, and slower timescales of change in bodily fluctuations and action (Liu et al., 2019). One plausible working hypothesis about the nature of within-trial dynamics is to presume dynamics similar to between-trial dynamics, rescaled to the faster timescale (e.g., Holden, 2013; Holden et al., 2014; Liu et al., 2019). Zooming in to very-fast timescales of neuronal variability, computational neuroscientists report that an important role of neuronal spiking behavior is to facilitate local synchronization and segregation among networks and local neuron populations of (Izhikevich, 2007; Damm et al., 2020). Furthermore, the organization of functional brain networks is often small-world, hierarchical, and loosely modular (Sporns, 2012). These core dynamical and physiological properties are sufficient to support spontaneous, evolving, and intermittent patterns of entrainment and resonance. Zooming out to times-scales slower than five-trial sequential effects often reveals a looser, and more variable form of entrainment, called 1/ƒ scaling (e.g., Gilden, 2001; Van Orden et al., 2003a, 2005; Diniz et al., 2011; Holden et al., 2011; Amon et al., 2018). Apparently, synchronization and entrainment are manifest over a broad range of timescales in cognition and action. This fact implies an essential role for oscillatory principles in the organization and maintenance of cognitive activity.

### Resonance

Historically, psychologists entertained resonance as a viable conceptual metaphor for recognition. Resonance was sometimes ascribed to hypothetical cognitive entities such as memory traces, or similar representational entities (e.g., Shepard, 1984; Neisser, 2014). However, other accounts were more circumspect about the material means for achieving resonance (e.g., Gibson and Carmichael, 1966; Grossberg and Stone, 1986; Treffner and Turvey, 1993; Turvey, 2007; Raja, 2020). The application of recurrent networks to problems in cognitive science offered a principled basis for the emergence of resonance in cognitive activity. For example, in the domain of word recognition, self-consistency refers to bidirectional, self-reinforcing activation that arises from coherent dynamic relations among spelling, phonology, and semantic nodes in recurrent networks (Stone and Van Orden, 1989, 1994; Van Orden et al., 1990; Kawamoto, 1993; Van Orden and Goldinger, 1994; Stone et al., 1997; Holden, 2002; Holden and Van Orden, 2002). Conceptually, self-consistency is equivalent to self-resonance in button-press dynamics. Recurrent systems operate on their own outputs in a manner akin to the notion of self-resonance described in the introduction – their dynamics are self-sustaining and self-reinforcing. Thus, recurrent dynamics imply an inherent capacity to support oscillatory entrainment. The coordinative interplay between participant and task across time is akin to an analogue recurrent system. For example, cooperative-competitive dynamics resembling mode-locking were used to explain articulatory, phonological, and semantically related errors that occur in word recognition performances, in which related errors are more likely, as when PINT is pronounced to rhyme with MINT (e.g., see Kawamoto, 1993; Van Orden and Goldinger, 1994; Kello and Plaut, 2000; Holden, 2002), or when ROWS is mistaken for a type of flower (e.g., Van Orden, 1987; Van Orden et al., 1999).

### Conclusions

The sequential effect results illustrate how intermittent, multiscale oscillatory entrainment offers a plausible route for biological systems to self-organize and coordinate goal directed cognitive activities over time (Van Orden and Holden, 2002; Holden and Rajaraman, 2012; Amon et al., 2018). Collectively, this multiscale, homeodynamic coordinative activity is commonly referred to as *interaction dominant dynamics* (e.g., Van Orden et al., 2003a).

The dynamic and physical principles governing coupled oscillators explained and controlled many classic empirical sequential effects. The grounding in natural law provided ample *a priori* predictions while avoiding detailed commitments to hypothetical cognitive entities or architectures. For instance, the expression of solo and dyadic sequential effects was quite similar, but the distributed nature of dyadic decisions renders them structurally distinct from solo decisions. Instead, oscillatory theory emphasizes a principled understanding of the informational couplings and dynamics governing an activity. It does so in terms of the interrelationships among individuals’ embodied histories and immediate environmental circumstances. Clearly, viable theoretical narratives regarding cognitive activity must respect these foundational constraints (Stephen and Van Orden, 2012).

The successful generalization of the HKB model to the details of sequential effects illustrates a tight coupling between perception, cognition, and action – mind and body. It also underscores a foundational role for oscillatory dynamics in the self-organization and persistence of goal directed behavior (Juarrero, 1999; Van Orden and Holden, 2002; Amon et al., 2018). The model revealed a prominent role for coordinative, nonlinear dynamics in the etiology of a long studied cognitive phenomenon and implicates coordinative relationships as important constraints in two-alternative forced-choice decision performance. Future research is expected to further clarify the relationship between oscillatory dynamics and cognitive performance.

## Data Availability Statement

The raw data supporting the conclusions of this article will be made available by the authors, without undue reservation.

## Ethics Statement

The studies involving human participants were reviewed and approved by University of Cincinnati IRB, California State University, Northridge IRB. Written informed consent for participation was not required for this study in accordance with the national legislation and the institutional requirements.

## Author Contributions

CA: editing, writing, data analysis, experimental design, data collection, and theoretical analysis. SF: editing, writing, data analysis, and editorial guidance. JH: editing, writing, data analysis, experimental design, theoretical development, and analysis. All authors contributed to the article and approved the submitted version.

### Conflict of Interest

The authors declare that the research was conducted in the absence of any commercial or financial relationships that could be construed as a potential conflict of interest.

## Footnotes

^1^https://youtu.be/qPeD87HJ0UA
